# Ultrasound-guided fascia iliaca compartment block versus intravenous analgesia in geriatric hip fractures: a systematic review and meta-analysis of randomized trials demonstrating superior pain control

**DOI:** 10.3389/fmed.2025.1611618

**Published:** 2025-10-14

**Authors:** Chang Liu, Shunyu Han, Ai Wei, Lina Yang, Mengchang Yang

**Affiliations:** ^1^Clinical Medical College, Southwest Medical University, Luzhou, China; ^2^Department of Anesthesiology, Chengdu Wenjiang District People’s Hospital, Chengdu, China; ^3^Qionglai Medical Center Hospital, Chengdu, China; ^4^Department of Anesthesiology, Sichuan Academy of Medical Sciences & Sichuan Provincial People's Hospital, Chengdu, China

**Keywords:** meta-analysis, RCT, FICB, intravenous analgesia, hip fractures

## Abstract

**Background:**

Severe pain in elderly hip fracture patients exacerbates perioperative risks. This meta-analysis compares ultrasound-guided fascia iliaca compartment block (UG-FICB) with intravenous analgesia for pain management.

**Methods:**

A comprehensive search of randomized controlled trials (RCTs) published through February 2025 was conducted across major databases, including English-language databases and Chinese databases. Outcomes analyzed via RevMan 5.3 using random/fixed-effect models. Primary outcomes encompassed Visual Analog Scale (VAS) scores, analgesic consumption, patient satisfaction, main adverse reactions, and gastrointestinal adverse events. Secondary outcomes included intraoperative blood loss, operative time, length of stay, and respiratory adverse events.

**Results:**

A total 26 RCTs (*n* = 2,347), UG-FICB significantly not only reduced Visual Analog Scale (VAS) scores at all timepoints: 0.5 h (*p* = 0.02), 2 h (*p* = 0.001), 4 h (*p* = 0.002), 6 h (*p* < 0.00001), 12 h (*p* = 0.0002), 24 h (*p* < 0.00001), and 48 h postoperatively (*p* = 0.003), but also reduced postoperative analgesic consumption (OR = 5.27, *p* < 0.00001). Patients receiving UG-FICB exhibited fewer drug-related adverse events, including dizziness (OR = 2.34), hypersomnia (OR = 3.58), and gastrointestinal complications (nausea OR = 2.57; constipation OR = 4.82; *p* < 0.05). UG-FICB also shortened length of stay (MD = 1.88 days, *p* < 0.00001) and enhanced satisfaction (OR = 0.26, *p* = 0.0002).

**Conclusion:**

Compared to intravenous analgesia, UG-FICB provides superior, sustained pain relief with fewer opioid-related complications and higher patient satisfaction. UG-FICB’s safety and efficacy advantages strongly support its adoption as first-line therapy in geriatric hip fractures protocols.

## 1 Introduction

Geriatric hip fractures (including femoral neck fractures and intertrochanteric fractures) represent a significant public health challenge in aging societies. Global data indicate that approximately 1.5 to 1.6 million geriatric patients sustain hip fractures annually (1, 2). The incidence demonstrates an exponential increase with advancing age. In China, the hip fracture incidence rate among populations aged ≥55 years ranges from 128.10 to 681.35 per 100,000 person-years. Global projections estimate the total caseload will surpass 6.3 million by 2050, with Asian demographics constituting over 50% of this disease burden (3). In the United States, the annual incidence of hip fractures among individuals aged ≥65 years approximates 100 cases per 1,000 population, with fracture rates demonstrating a strong age-dependent correlation that manifests as significantly elevated incidence rates in populations aged ≥70 years (4, 5). These patients face substantial postoperative mortality (one-year rates: 14–27.3%), further amplified by complications such as deep vein thrombosis, surgical site infections, and major cardiovascular events (6–8). Thus, optimizing pain management is critical for risk mitigation.

Intravenous analgesia serves as the primary modality for traditional postoperative pain management, offering advantages such as rapid onset of action, ease of administration, and the capacity for individualized dose titration through patient-controlled intravenous analgesia (9). However, older patients are particularly susceptible to opioid accumulation due to age-related declines in hepatic and renal function, which may precipitate adverse effects including respiratory depression, nausea and vomiting, excessive sedation, and delirium (with an incidence rate as high as 25%) (10, 11). UG-FICB has been demonstrated to effectively mitigate the risk of opioid-associated adverse events (12). However, UG-FICB faces significant practical limitations: it demands advanced sonographic skills and anatomical proficiency; necessitates accessible ultrasound instruments; exhibits variable efficacy with increased failure rates in obese patients (BMI > 35 kg/m^2^) or those with anatomical anomalies where fascial planes are obscured (13). Critically, despite emerging alternatives like PENG block, suprainguinal FICB (the comparator technique in this meta-analysis) maintains widespread adoption given its consistent efficacy and technical accessibility under ultrasound guidance (12).

Despite growing clinical adoption of regional anesthesia techniques, contemporary analgesic protocols for geriatric hip fractures demonstrate a critical evidence void: there remains a paucity of comprehensive systematic reviews comparing UG-FICB with standard intravenous analgesia. This systematic review undertakes a rigorous comparative analysis of analgesic efficacy, opioid-sparing effects, and complication profiles between these modalities.

## 2 Materials and methods

### 2.1 Study design

This systematic review with meta-analysis was conducted in accordance with the PRISMA 2020 (Preferred Reporting Items for Systematic Reviews and Meta-Analyses) statement (14). The protocol adhered to Cochrane Handbook for Systematic Reviews of Interventions standards.

### 2.2 Literature retrieval strategy

Boolean searches were executed across English-language (Cochrane Library, Embase, PubMed, Scopus, Web of Science) and Chinese databases (CNKI, VIP, Wan Fang) through February 2025. We conducted to manually search the bibliographies of RCTs comparing UG-FICB and standard intravenous analgesia. Our search strategy incorporated both subject headings and free-text keywords: (“hip fracture” OR “pertrochanteric fracture”) AND (“fascia iliaca block” OR “FICB” OR “regional anesthesia”) AND (“ultrasound guidance” OR “ultrasonography”) AND (“elderly” OR “geriatric”). This literature retrieval strategy is detailed in Supplementary File 1.

### 2.3 Eligibility criteria (PICOS framework)

Population: Patients ≥65 years with hip fracture (including femoral neck fractures and intertrochanteric fractures) requiring surgical intervention, excluding pathological fractures or polytrauma cases.
Intervention: Preoperative ultrasound-guided FICB.
Comparison: Standard intravenous opioid analgesia with/without PCA.
Outcomes: Comparing pain score VAS at 0.5/2/4/6/12/24/48 h. Opioid consumption, length of stay, operation time, incidence of drug-related adverse events, and so on.
Study design: Only including RCTs.

### 2.4 Data extraction & quality assessment

Two independent reviewers performed blinded data extraction using structured electronic data extraction forms (Cochrane Collaboration standardized template). Any disagreements between the reviewers were resolved by consultation with a third reviewer. All included study literature was collected based on outcome measures (Author, publication year, sample size, age stratification, ASA classification, surgery, and so on).

The assessment of bias was performed utilizing the Cochrane RoB 2.0 (Seven-domain evaluation: Randomization process; Allocation sequence generation; Participant and personnel blinding; Missing outcome data; Outcome measurement (blinded assessment); Selective reporting; Potential other biases). The risk of bias assessment (“low risk of bias,” “unclear risk of bias,” or “high risk of bias”) (15) for the aforementioned seven items was independently evaluated by two researchers. Any discrepancies in assessment were adjudicated by a senior biomedical expert. To address incomplete reporting of methodological details (such as: randomization protocols, allocation concealment mechanisms, and blinding procedures), corresponding authors of included studies were contacted via email to request missing information. However, no additional methodological clarifications were obtained.

### 2.5 Statistical analysis

Statistical analysis was performed using RevMan 5.4 software (freely available online). Dichotomous variables were calculated as odds ratios (OR) with 95% confidence intervals (95% CI), while continuous variables were quantified using mean differences (MD) or standardized mean difference (SMD) with 95% CI. Heterogeneity among included studies was assessed through the following criteria: studies demonstrating *p* ≥ 0.1 and I^2^ ≤ 50% were considered homogeneous and analyzed using a fixed-effect model. When substantial heterogeneity was identified (I^2^ > 50% or *p* < 0.1), a random-effects model was employed. This approach accounts for both within-study sampling error and between-study variance in effect sizes, acknowledging that clinical diversity (such as: variations in UG-FICB techniques, anesthetic dosing, patient comorbidities) and methodological differences (such as: blinding limitations, outcome assessment protocols) likely contribute to true variation in underlying effects. The random-effects model provides more conservative confidence intervals, reducing the risk of overestimating precision when heterogeneity is high.

Clinical outcome assessments were rigorously conducted according to the GRADE framework. For all included RCTs, predefined downgrading criteria were systematically applied: a 1-level downgrade when risk ratios’ 95% CI crossed the null line, with additional “serious” imprecision downgrades for study arms with <50 participants in pooled analyses. Two reviewers independently performed GRADE quality ratings, with discrepancies resolved through iterative discussions until consensus was achieved.

## 3 Results

### 3.1 Search result and study characteristic

**Figure 1:**
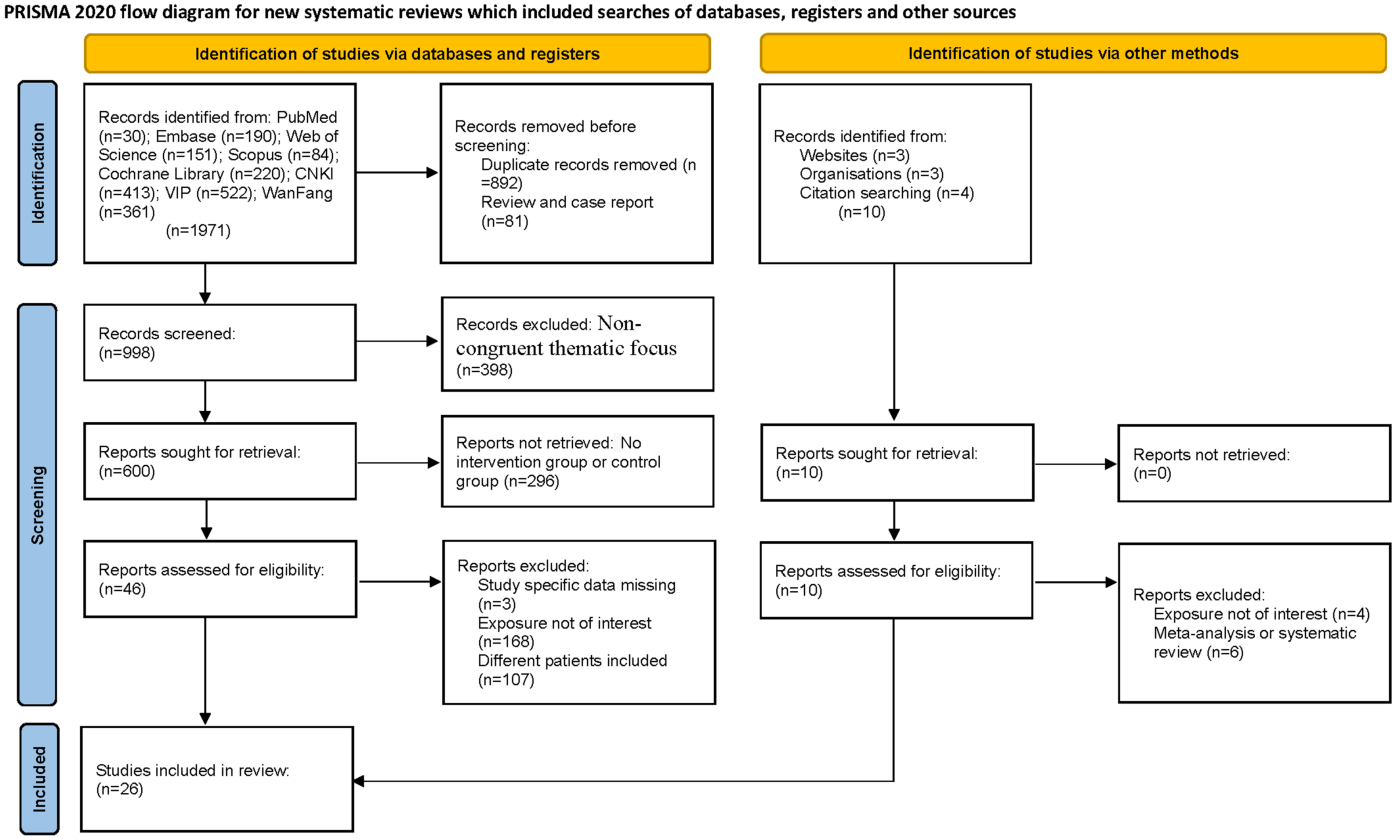
The flowchart of the study.

The systematic search encompassed eight major databases, identifying 1,981 potentially relevant articles published between 1990 and 2025. Initial exclusion of 892 duplicate records was performed using EndNote software. Subsequent title/abstract screening eliminated 81 non-eligible publications (reviews and case reports). Full-text evaluation of the remaining 1,008 articles resulted in exclusion of 398 studies due to non-congruent thematic focus, 168 studies for divergent intervention modalities, and 107 studies with non-conforming patient populations. Ultimately, 26 articles (16–41) met our inclusion criteria for qualitative synthesis. Figure 1 presents the PRISMA-compliant selection flowchart.

A total of 26 RCTs comparing UG-FICB with standard intravenous analgesia for analgesic efficacy in elderly patients with hip fractures were included. These 26 studies enrolled 2,347 participants collectively. The sample sizes of the included studies ranged from 22 to 178 cases, with fracture types encompassing femoral fractures, intertrochanteric fractures, and femoral neck fractures. Participants underwent either internal fixation surgery or hip arthroplasty. One study lacked age information of participants, 11 studies did not specify ASA (American Society of Anesthesiologists) classification, and 2 studies failed to report participants’ gender. The baseline characteristics of the included studies are detailed in Table 1.

**Table 1:** Basic characteristics of the included literature.

Name	Year	Study type	Population	Age (I/C)	Number of persons (I/C)	Intervention group	Control group	Outcome measures
Ali. FA	2024	RCT	Acetabular fractures	42.5/35.7	10/10	S-FICB	Intravenous analgesia	1, VAS Score; 2, Total Opioid Consumption; 3, Complications
Thompson. J	2019	RCT	Fractures of the proximal femur	NA	23/24	FICB	Intravenous analgesia	1, The total consumption of analgesics; 2, Patient Satisfaction
Yamamoto. N	2019	RCT	Hip fracture	84.7/84.6	25/28	FICB	Intravenous analgesia	1, VAS score; 2, The total number of rescue analgesics; 3, Delirium occurring 4, potential drug or block-related complications
Morrison. RS	2016	RCT	Hip fracture	83.4/81.7	72/81	FICB	Intravenous analgesia	1, Pain Scores; 2, Opioid-related Conditions
Bang. S	2016	RCT	Femoral fracture	81.6/82.0	11/11	FICB+PICA	Intravenous analgesia	1, Pain Scores; 2, Postoperative Complications; 3, Use of Additional Analgesics
Mostafa. SF	2018	RCT	Femoral fracture	59.70/58.43	30/30	FICB	Intravenous analgesia	1, Postoperative VAS Score; 2, Use of Postoperative Rescue Analgesics; 3, Intraoperative Fentanyl Consumption; 4, Postoperative Complications; 5, Patient Satisfaction and Sedation Scores
KHAN. MH	2021	RCT	Hip fracture	57.17/58.25	40/40	FICB	Intravenous analgesia	1, Pain Scores; 2, Patient Satisfaction; 3, Postoperative Complications
Chen. T	2023	RCT	Lower limb fracture	65.4/65.9	40/40	S-FICB	Intravenous analgesia	1, VAS Pain Score; 2, Tramadol Analgesic Rescue Situation; 3, Analgesic Satisfaction Score; 4, Incidence of Adverse Reactions
Shan. ZB	2020	RCT	Hip fracture	68.2/67.2	30/30	FICB+PICA	Intravenous analgesia	1, VAS Pain Score; 2, Incidence of Respiratory Depression; 3, Operation Time; 4, Completion Time
Han. CZ	2022	RCT	Hip fracture	70.19/71.04	61/60	FICB	Intravenous analgesia	1, Adverse Reactions
Wang. JL	2023	RCT	Femoral fracture	74.45/74.26	52/52	FICB	Intravenous analgesia	1, Operation time; 2, Intraoperative blood loss
Ning. JH	2015	RCT	Femoral fracture	69/68	60/60	FICB	Intravenous analgesia	1, VAS Pain Score; 2, Toxicity Reactions
Piao. HW	2020	RCT	Hip fracture	77.23/76.75	60/60	FICB	Intravenous analgesia	1, VAS Pain Score; 2, Postoperative Complications; 3, Adverse Reactions
Qing. LP	2018	RCT	Proximal femur fracture	72.14/72.58	50/50	FICB	Intravenous analgesia	1, VAS Pain Score; 2, The incidence of complications
Shen. Y	2021	RCT	Hip fracture	71.4/70.9	40/40	FICB	Intravenous analgesia	1, VAS Pain Score; 2, The incidence of complications
Sun. QQ	2021	RCT	Hip fracture	76.5/77.1	40/40	FICB	Intravenous analgesia	1, VAS Pain Score; 2, The incidence of complications
Tan. ZQ	2019	RCT	Hip fracture	70.7/70.4	64/64	FICB	Intravenous analgesia	1, VAS Pain Score; 2, The incidence of complications
Wang. WT	2020	RCT	Femoral intertrochanteric fracture	73/72	30/30	FICB	Intravenous analgesia	1, VAS Pain Score; 2, The incidence of complications
Xie. J	2024	RCT	Hip fracture	75.9/76.9	40/40	S-FICB	Intravenous analgesia	1, VAS Pain Score; 2, The incidence of complications
Xu. TS	2021	RCT	Hip fracture	86.37/85.92	89/89	FICB	Intravenous analgesia	1, VAS Pain Score; 2, The incidence of complications; 3, Operation time
Xu. Z	2020	RCT	Hip fracture	75.3/76.2	61/60	FICB	Intravenous analgesia	1, The incidence of complications
Xu. XX	2023	RCT	Elderly patients with hip fracture	70.52/70.58	52/51	FICB	Intravenous analgesia	1, VAS Pain Score; 2, The incidence of complications
Yao. F	2024	RCT	Hip replacement for hip fracture	69.68/70.43	42/42	FICB+PCIA	Intravenous analgesia	1, VAS Pain Score; 2, The incidence of complications
Yao. MF	2019	RCT	Elderly patients with hip fracture	81.3/80.1	67/67	FICB	Intravenous analgesia	1, VAS Pain Score; 2, The incidence of complications
Li. CX	2023	RCT	Elderly patients with hip fracture	82.4/81.4	62/65	FICB	Intravenous analgesia	1, VAS Pain Score; 2, The incidence of complications
Guo. JW	2021	RCT	Elderly patients with femoral intertrochanteric fracture	75.13/76.39	36/36	FICB	Intravenous analgesia	1, VAS Pain Score; 2, The incidence of complications; 3, Operation time

FICB, fascia iliaca compartment block; S-FICB, supra-inguinal fascia iliaca compartment block; PCIA, patient controlled intravenous analgesia; NA, not available; VAS, Visual Analog Scale.

### 3.2 Bias risk assessment

**Figure 2:**
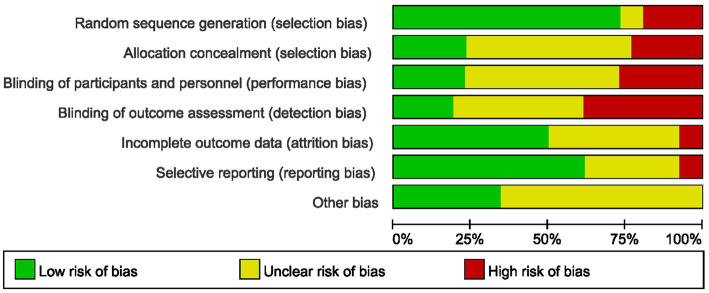
Results of quality assessment using the Cochrane risk tool.

The methodological rigor of included RCTs was critically appraised using Cochrane RoB 2.0. Key concerns arose from inadequate reporting. Random sequence generation: Only 19/26 studies described sequence generation methods; 7 omitted or ambiguously reported this (high risk of selection bias). Among these, 5 used non-random methods, introducing confounding. Allocation concealment: 20 studies (77%) failed to report concealment mechanisms, enabling selection bias. 6 studies described concealment mechanisms. Blinding: 6 studies documented blinding protocols, 13 studies provided insufficient details, and 7 studies lacked any description of blinding procedures. Incomplete outcome: 2 studies exhibited incomplete outcome reporting, 13 studies demonstrated complete outcome data, while 11 studies did not specify data completeness. Selective reporting: 2 studies showed evidence of selective reporting, 16 studies were free from selective reporting, and 8 studies had insufficient information to permit judgment. Overall, 18 studies (69%) exhibited “high risk” or “some concerns” across ≥3 domains (Figure 2). These deficiencies may inflate UG-FICB’s perceived efficacy, particularly for patient-reported outcomes. GRADE methodology, with comprehensive documentation provided in Supplementary File 2. The overall certainty of evidence was categorized as moderate to very low.

### 3.3 Primary results

#### 3.3.1 VAS scores

**Figure 3:**
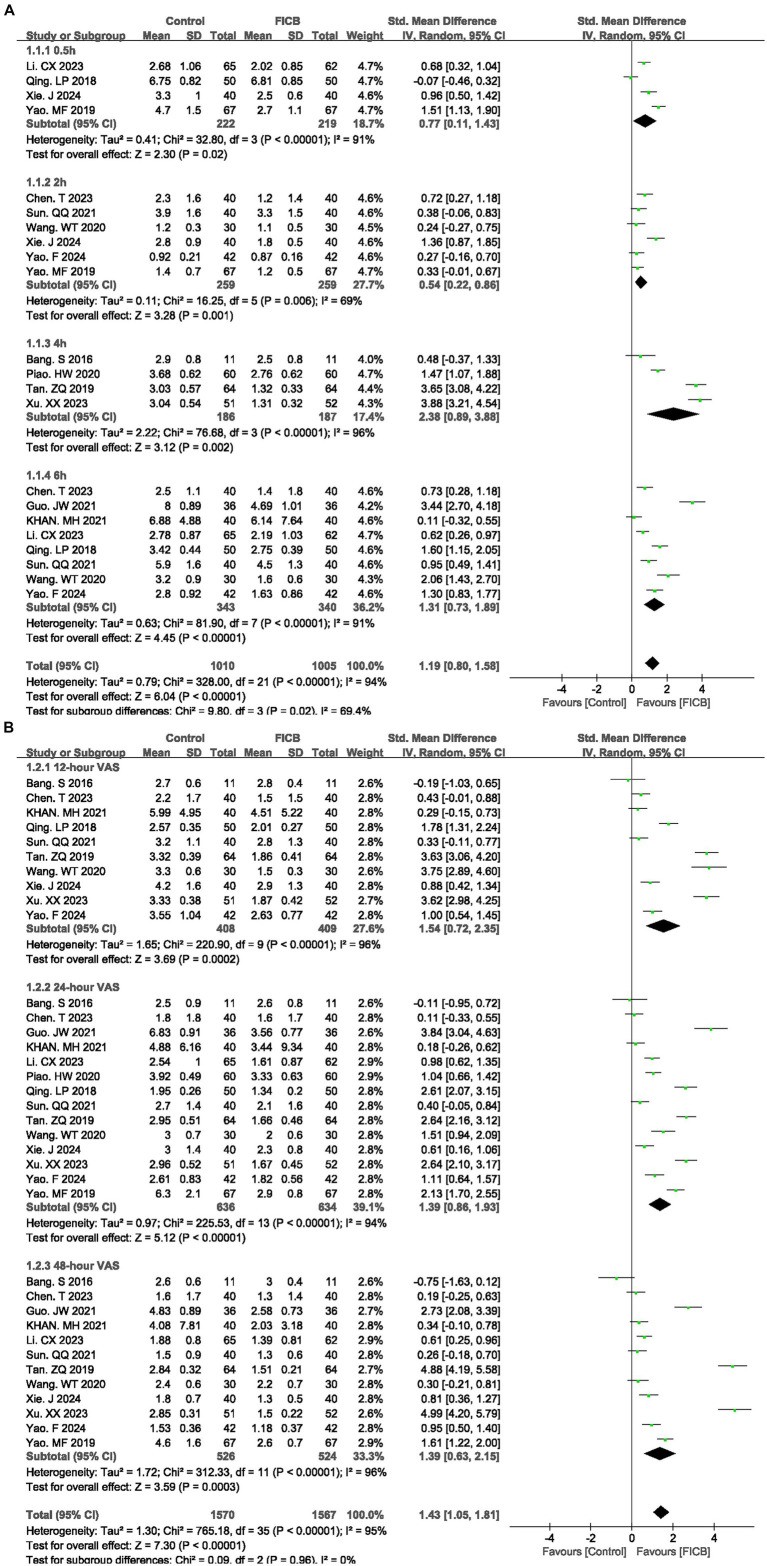
**(A,B)** A forest plot showing the VAS.

Fourteen studies (17–19, 21, 22, 26, 27, 30, 31, 34, 35, 37, 40, 41) reported VAS scores at multiple time points (0.5 h, 2 h, 4 h, 6 h, 12 h, 24 h, and 48 h). Due to significant heterogeneity among studies (*p* < 0.00001, *I^2^* = 94%), a random-effects model was employed for meta-analysis. The diamond icon to the right of the midline, concurrently labeled “Favours FICB”, and a confidence interval not exceeding “0” indicates that the difference is statistically significant (*p* < 0.05). Forest plots demonstrated statistically significant VAS reductions favoring UG-FICB at all timepoints (0.5 h: SMD = 0.77, 95% CI: 0.11 to 1.43; *p* = 0.02; 2 h: SMD = 0.54, 95% CI: 0.22 to 0.86; *p* = 0.001; 4 h: SMD = 2.38, 95% CI: 0.89 to 3.88; *p* = 0.002; 6 h: SMD = 1.31, 95% CI: 0.73 to 1.89; *p* < 0.00001; Figure 3A) (12 h: SMD = 1.54, 95% CI: 0.72 to 2.35; *p* = 0.0002; 24 h: SMD = 1.39, 95% CI: 0.86 to 1.93; *p* < 0.00001; 48 h: SMD = 1.39, 95% CI: 0.63 to 2.15; *p* = 0.0003; Figure 3B). Subsequently, sensitivity analyses were conducted to explore potential sources of heterogeneity; however, these analyses failed to identify definitive contributors to the observed heterogeneity. In the quality assessment of included studies, key methodological details such as randomization and blinding protocols were predominantly graded as “unclear.” Additionally, incomplete reporting of critical baseline characteristics—including age distributions, fracture types, and surgical approaches—may constitute potential sources of heterogeneity. Concurrently, variations in FICB technical parameters (drug selection, administered dosage, and infusion duration) likely contributed to increased heterogeneity. Outcome level quality for VAS scores at multiple time points (0.5 h, 2 h, 4 h, 6 h, 12 h, 24 h, and 48 h) assessed by GRADE were “very low.”

#### 3.3.2 Analgesics

**Figure 4:**
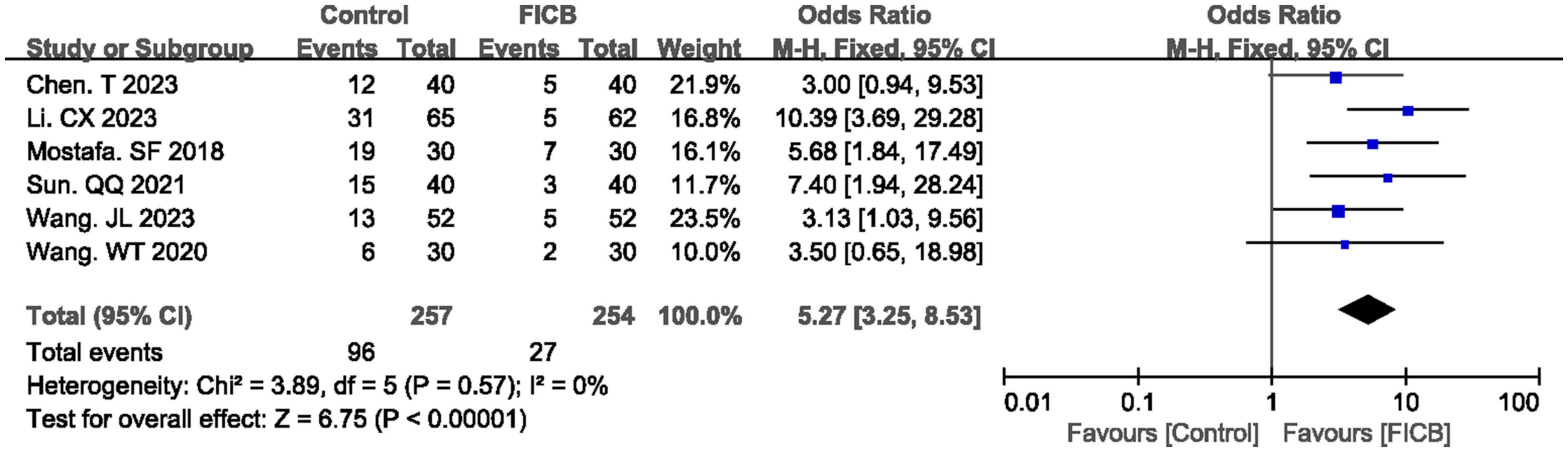
A forest plot showing the analgesics.

Six studies (18, 22, 24, 30, 33, 34) reported postoperative analgesic requirement rates, while three studies (24, 33, 40) documented analgesic dosage. No significant heterogeneity was observed in either outcome (postoperative analgesic requirement rate: *p* = 0.57, *I*^2^ = 0%; analgesic dosage: *p* = 0.79, *I*^2^ = 0%), justifying the use of a fixed-effect model. The diamond icon to the right of the midline, concurrently labeled “Favours FICB”, and a confidence interval not exceeding “0” or “1” indicates that the difference is statistically significant (*p* < 0.05). Meta-analysis revealed that the FICB group demonstrated both a lower postoperative analgesic requirement rate (OR = 5.27, 95% CI: 3.25 to 8.53; *p* < 0.00001, Figure 4) and reduced analgesic dosage (MD = 7.79, 95% CI: 5.67 to 9.91; *p* < 0.00001, Supplementary File 3; Supplementary Figure S1) compared to the intravenous analgesia group. Outcome level quality for analgesic requirement rate and reduced analgesic dosage assessed by GRADE was “Moderate.”

#### 3.3.3 Satisfaction

**Figure 5:**
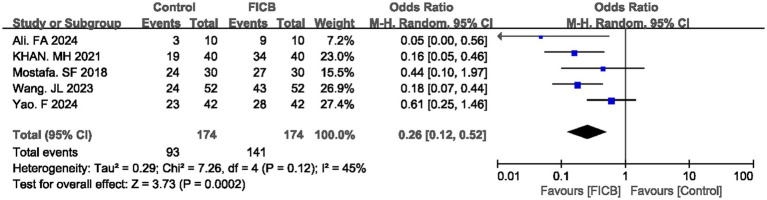
A forest plot showing the satisfaction.

Five studies (16, 21, 24, 33, 40) reported satisfaction rate. No significant heterogeneity was observed in either outcome (*p* = 0.12, *I*^2^ = 45%), justifying the use of a fixed-effect model. The diamond icon to the right of the midline, concurrently labeled “Favours FICB”, and a confidence interval not exceeding “1” indicates that the difference is statistically significant (*p* < 0.05). Meta-analysis revealed that the FICB group demonstrated both a higher satisfaction rate (OR = 0.26, 95% CI: 0.12 to 0.52; *p* = 0.0002, Figure 5) compared to the intravenous analgesia group. Outcome level quality for satisfaction rate assessed by GRADE was “Moderate.”

#### 3.3.4 Main adverse reactions

**Figure 6:**
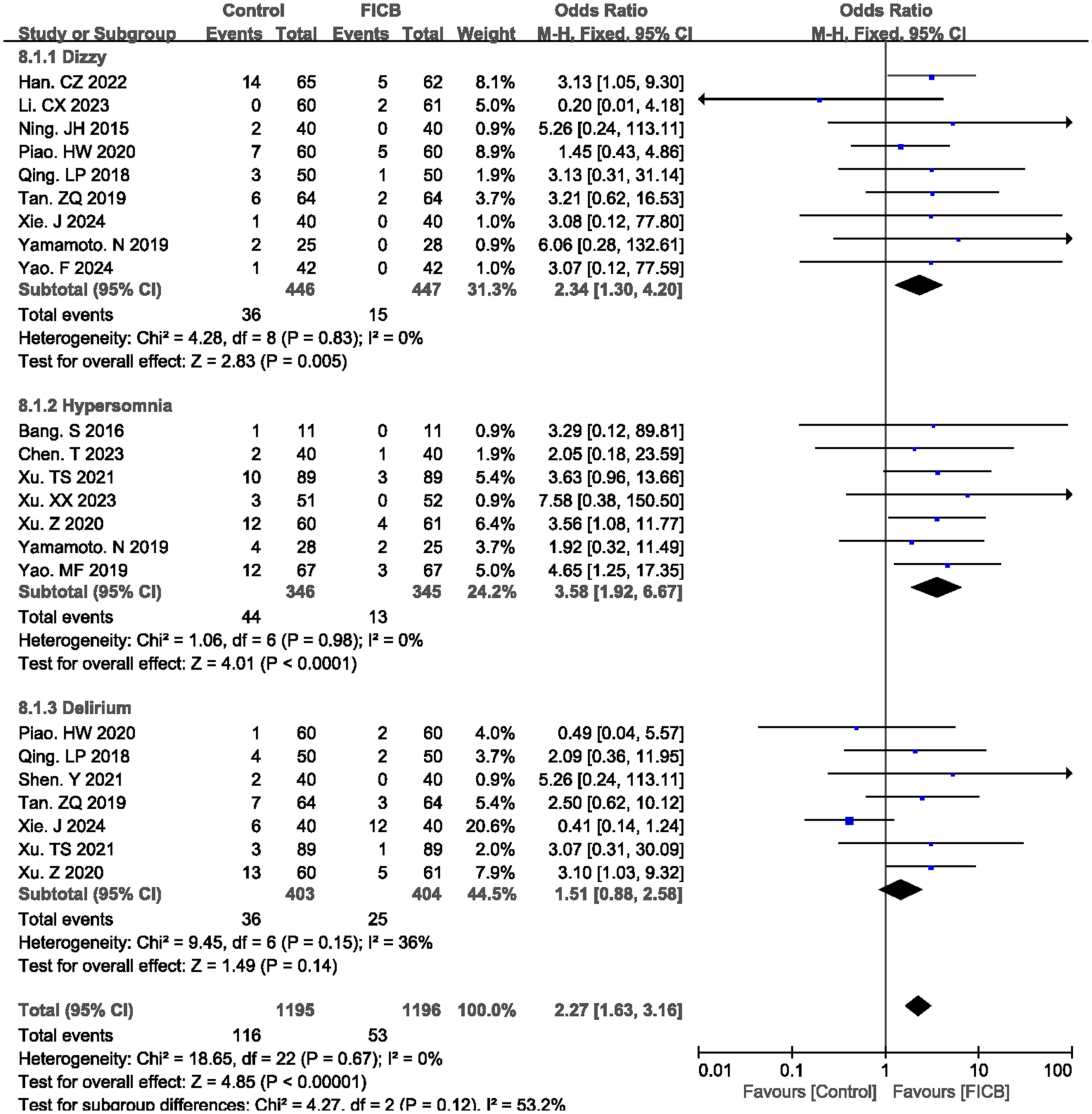
A forest plot showing the main adverse reactions.

Nine studies (20, 22, 25–27, 31, 35, 39, 40) reported postoperative dizzy rate, seven studies (17, 18, 36–39, 41) reported postoperative hypersomnia rate, and seven studies (26, 27, 29, 31, 35, 36, 38) reported postoperative delirium rate. No significant heterogeneity was observed (dizzy: *p* = 0.83, *I*^2^ = 0%; hypersomnia: *p* = 0.98, *I*^2^ = 0%; delirium: *p* = 0.15, *I*^2^ = 0%), justifying the use of a fixed-effect model. The diamond icon to the right of the midline, concurrently labeled “Favours FICB”, and a confidence interval not exceeding “1” indicates that the difference is statistically significant (*p* < 0.05). However, for the postoperative delirium rate, the diamond icon is placed on the midline, and the confidence interval crosses “1,” indicating that there is no statistical significance in the postoperative delirium rate between the intravenous analgesia group and the FICB group. Meta-analysis revealed that the FICB group demonstrated both a lower postoperative dizzy rate (OR = 2.34, 95% CI: 1.30 to 4.20; *p* = 0.005, Figure 6) and hypersomnia rate (OR = 3.58, 95% CI: 1.92 to 6.67; *p* < 0.0001, Figure 6) compared to the intravenous analgesia group. But for postoperative delirium rate, two groups had no significant difference (OR = 1.51, 95% CI: 0.88 to 2.58; *p* = 0.14, Figure 6). Outcome level quality for postoperative dizzy rate, hypersomnia rate, and delirium rate assessed by GRADE were “Moderate.”

#### 3.3.5 Gastrointestinal adverse

**Figure 7:**
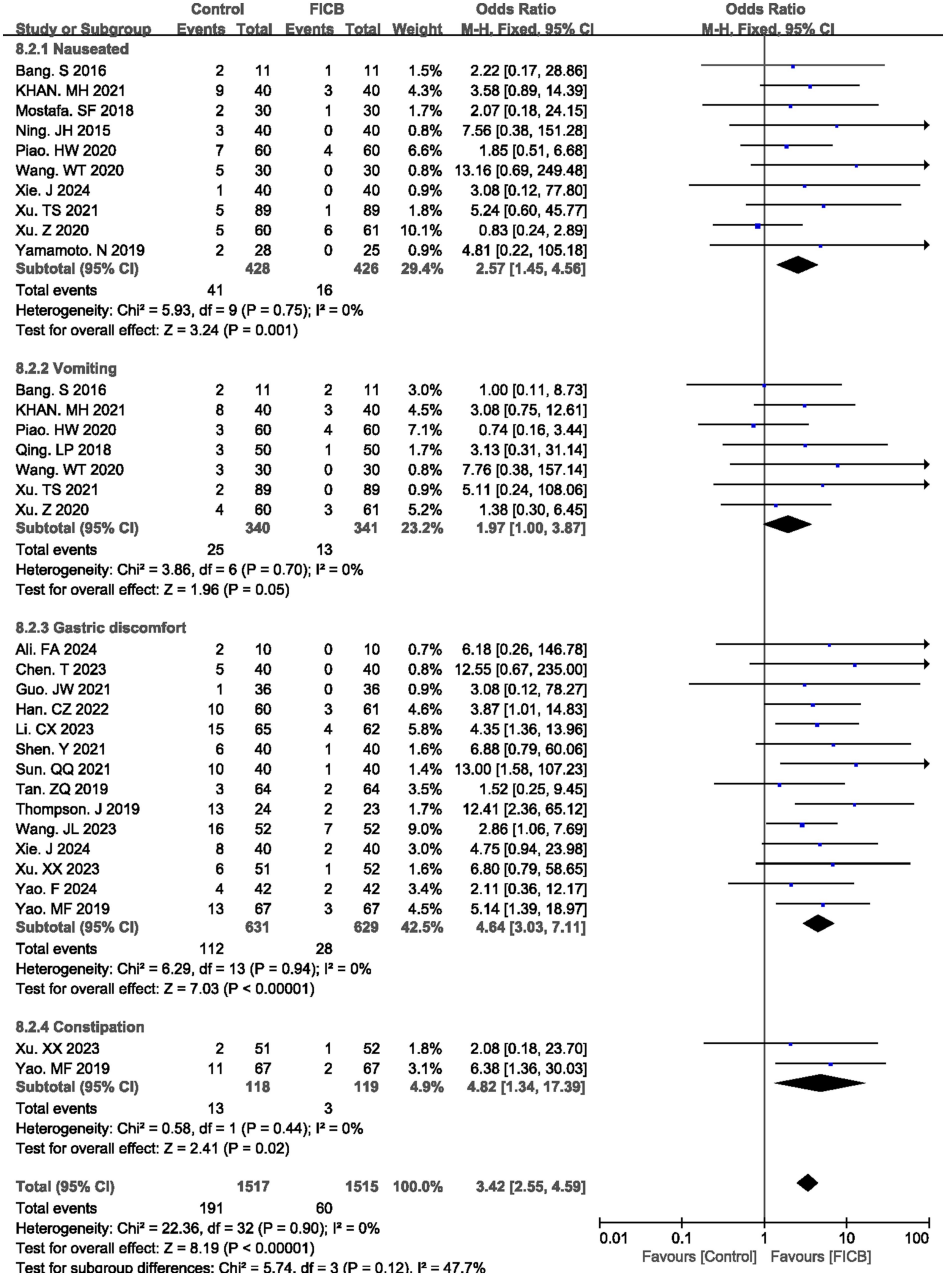
A forest plot showing the gastrointestinal adverse.

The meta-analysis evaluated gastrointestinal adverse effects associated with pharmacologic interventions, comparing the FICB group with the intravenous analgesia group across four outcomes: nausea, vomiting, gastric discomfort, and constipation. Ten studies (17, 21, 24–26, 34–36, 38, 39) reported postoperative nausea rates, seven studies (17, 21, 26, 27, 34, 36, 38) documented vomiting incidence, fourteen studies (16, 18–20, 22, 29–33, 35, 37, 40, 41) described gastric discomfort, and two studies (37, 41) quantified constipation rates. All outcomes demonstrated homogeneity across studies (nausea: *p* = 0.75, *I^2^* = 0%; vomiting: *p* = 0.70, *I^2^* = 0%; gastric discomfort: *p* = 0.94, *I^2^* = 0%; constipation: *p* = 0.90, *I^2^* = 0%), supporting the application of a fixed-effect model. The diamond icon to the right of the midline, concurrently labeled “Favours FICB”, and a confidence interval not exceeding “1” indicates that the difference is statistically significant (*p* < 0.05). Pooled analysis revealed statistically significant reductions in the FICB group for all gastrointestinal outcomes compared to intravenous analgesia (nausea: OR = 2.57, 95% CI: 1.45 to 4.56, *p* = 0.001; vomiting: OR = 1.97, 95% CI: 1.00 to 3.87, *p* = 0.05; gastric discomfort: OR = 4.64, 95% CI: 3.03 to 7.11, *p* < 0.00001; constipation: OR = 4.82, 95% CI: 1.34 to 17.39, *p* = 0.02), with consolidated results visualized in Figure 7. Outcome level quality for nausea, vomiting, gastric discomfort, and constipation assessed by GRADE were “Moderate.”

### 3.4 Secondary results

#### 3.4.1 Interoperative bleeding loss

Four studies (17, 33, 36, 40) reported interoperative bleeding loss. No significant heterogeneity was observed (*p* = 0.54, *I*^2^ = 0%), justifying the use of a fixed-effect model. The diamond icon is placed on the midline, and the confidence interval crosses “0,” indicating that there is no statistical significance in the interoperative bleeding loss between the intravenous analgesia group and the FICB group. Meta-analysis revealed that the FICB group was no superior to intravenous analgesia group (MD = 0.45 mL, 95% CI: −0.79 to 1.70; *p* = 0.48, Supplementary File 3; Supplementary Figure S2). Outcome level quality for interoperative bleeding loss assessed by GRADE were “Moderate.”

#### 3.4.2 Operative time

Ten studies (17–19, 23, 27, 30, 33, 34, 36, 40) reported operative time. No significant heterogeneity was observed (*p* = 0.53, *I*^2^ = 0%), justifying the use of a fixed-effect model. The diamond icon is placed on the midline, and the confidence interval crosses “0,” indicating that there is no statistical significance in the operative time between the intravenous analgesia group and the FICB group. Meta-analysis revealed that the FICB group was no superior to intravenous analgesia group (SMD = -0.08, 95% CI: −0.21 to 0.05; *p* = 0.24, Supplementary File 3; Supplementary Figure S3). Outcome level quality for operative time assessed by GRADE were “Low.”

#### 3.4.3 Length of stay

Thirteen studies (19, 22, 23, 27, 31, 33–38, 40, 41) reported length of stay. Due to significant heterogeneity among studies (*p* < 0.00001, *I^2^* = 94%), a random-effects model was employed for meta-analysis. The diamond icon to the right of the midline, concurrently labeled “Favours FICB”, and a confidence interval not exceeding “0” indicates that the difference is statistically significant (*p* < 0.05). Meta-analysis revealed that the FICB group was superior to intravenous analgesia group (MD = 1.88 days, 95% CI: 1.09 to 2.67; *p* < 0.00001, Supplementary File 3; Supplementary Figure S4). Therefore, compared with intravenous analgesia, the FICB group had a shorter length of stay. Subsequently, sensitivity analyses were conducted to explore potential sources of heterogeneity; however, these analyses failed to identify definitive contributors to the observed heterogeneity. The heterogeneity in length of stay may originate from three principal sources: variations in surgeon procedural proficiency, fundamental differences in fracture patterns and corresponding surgical interventions, and disparate postoperative rehabilitation. Outcome level quality for length of stay assessed by GRADE were “Very low.”

#### 3.4.4 Respiratory adverse

The meta-analysis evaluated respiratory adverse effects associated with pharmacologic interventions, comparing the FICB group with the intravenous analgesia group across two outcomes: respiratory depression and pulmonary infection. Three studies (16, 18, 28) reported postoperative respiratory depression rates, and four studies (19, 22, 26, 33) documented pulmonary infection incidence. All outcomes demonstrated homogeneity across studies (*p* = 0.68, *I^2^* = 0% and *p* = 0.51, *I^2^* = 0%), supporting the application of a fixed-effect model. The diamond icon to the right of the midline, concurrently labeled “Favours FICB”, and a confidence interval not exceeding “1” indicates that the difference is statistically significant (*p* < 0.05). Pooled analysis revealed statistically significant reductions in the FICB group for the both outcomes compared to intravenous analgesia (OR = 7.11, 95% CI: 1.25 to 40.61, *p* = 0.03 and OR = 2.30, 95% CI: 1.14 to 4.68, *p* = 0.002), with consolidated results visualized in Supplementary File 3 and Supplementary Figure S5. Outcome level quality for respiratory depression and pulmonary infection assessed by GRADE were “Low” and “Moderate.”

#### 3.4.5 Other adverse

Eight studies (19, 22, 26, 27, 31, 33, 37, 41) reported postoperative thrombosis rate, five studies (24, 26, 31, 33, 35) reported postoperative cardiovascular accident rate, and three studies (17, 21, 40) reported postoperative pruritus rate. No significant heterogeneity was observed (thrombosis: *p* = 0.85, *I*^2^ = 0%; cardiovascular accident: *p* = 0.89, *I*^2^ = 0%; pruritus: *p* = 1.00, *I*^2^ = 0%), justifying the use of a fixed-effect model. The diamond icon to the right of the midline, concurrently labeled “Favours FICB”, and a confidence interval not exceeding “1” indicates that the difference is statistically significant (*p* < 0.05). However, for the postoperative cardiovascular accident rate and pruritus rate, the diamond icon is placed on the midline, and the confidence interval crosses “1,” indicating that there is no statistical significance in the postoperative cardiovascular accident rate and pruritus rate between the intravenous analgesia group and the FICB group. Meta-analysis revealed that the FICB group demonstrated both a lower postoperative thrombosis rate (OR = 2.56, 95% CI: 1.53 to 4.28; *p* = 0.0003, Supplementary File 3; Supplementary Figure S6) compared to the intravenous analgesia group. But for postoperative cardiovascular accident rate and pruritus rate, two groups had no significant difference (OR = 1.48, 95% CI: 0.80 to 2.74; *p* = 0.22, and OR = 3.29, 95% CI: 0.85 to 12.71; *p* = 0.08, Supplementary File 3; Supplementary Figure S6). Outcome level quality for postoperative thrombosis rate, cardiovascular accident rate, and pruritus rate assessed by GRADE was “Moderate.”

### 3.5 Publication bias

**Figure 8:**
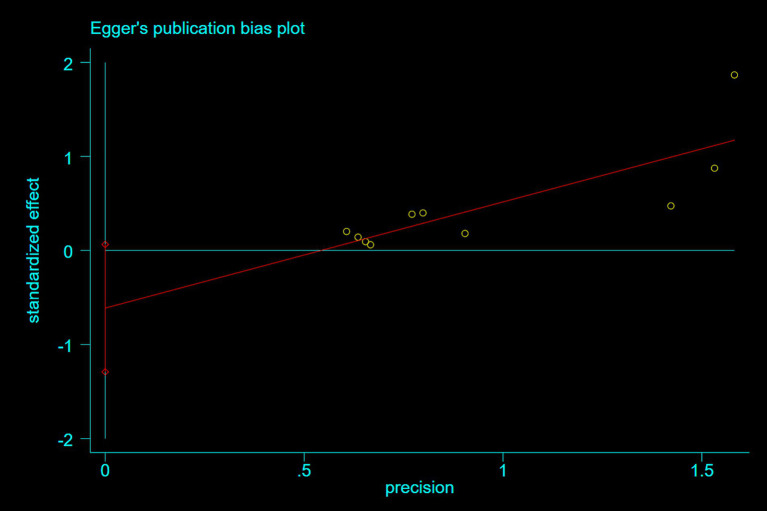
A plot showing the publication bias for nausea.

**Figure 9:**
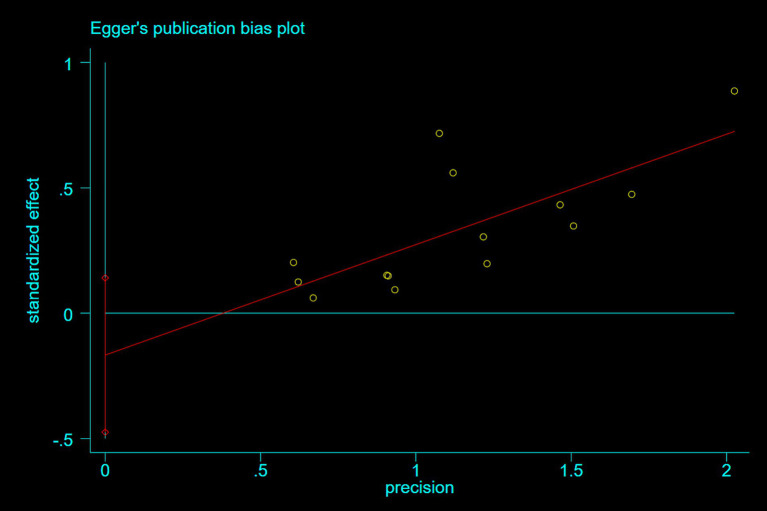
A plot showing the publication bias for gastric discomfort.

Publication bias was assessed using Egger’s test. For studies reporting nausea and epigastric distress, Egger’s tests demonstrated symmetrical funnel distributions (Figure 8: *p* = 0.07; Figure 9: *p* = 0.26), indicating no significant publication bias. Moreover, supplementary analyses of length of stay and operative duration similarly failed to reveal publication bias (Supplementary File 3; Supplementary Figure S7: *p* = 0.184; Supplementary Figure S8: *p* = 0.654).

## 4 Discussion

### 4.1 Epidemiological background and clinical necessity

Hip fractures pose a significant global public health challenge, with an annual incidence exceeding 1.6 million cases, predominantly affecting individuals aged 65 years and older (accounting for >80% of total cases) (42, 43). Population aging will likely increase this burden to 6.3 million annual cases by 2050 (44). These fractures often precipitate rapid health deterioration, with 1-year post-fracture mortality rates ranging from 14 to 36%, and studies indicate an 8-fold increase in mortality risk within 3 months post-fracture (45). Life-threatening complications such as pneumonia, venous thromboembolism (encompassing deep vein thrombosis and pulmonary embolism), and delirium further exacerbate clinical risks (46). Effective pain management is paramount in this population, as uncontrolled pain amplifies immobility-related complications, delirium incidence, and systemic inflammatory responses (47, 48).

First described by Dalens et al. (49), FICB is a regional anesthesia technique for perioperative analgesia in hip, femoral, and knee surgeries. Local anesthetic injection into the fascia iliaca compartment achieves anterior hip and lateral thigh analgesia by targeting the femoral, lateral femoral cutaneous, and obturator nerves (49). A Cochrane review demonstrated that FICB significantly reduces pain scores, opioid consumption, and secondary outcomes such as delirium incidence, hospital length of stay, and pneumonia rates (50). Traditional intravenous analgesia remains the cornerstone of perioperative analgesia, predominantly utilizing opioids. While offering rapid onset and dose flexibility, age-related declines in hepatic/renal function predispose elderly patients to delayed drug metabolism, active metabolite accumulation, and dose-dependent risks: respiratory depression (15–30%) and gastrointestinal hypomotility (51). Furthermore, *μ*-opioid receptor-mediated inhibition of enteric nervous activity results in constipation rates exceeding 60% (52). These effects disproportionately impact frail geriatric populations with diminished physiological reserve (53). Currently, no systematic evaluation comprehensively compares the efficacy and safety profiles of regional versus intravenous analgesia.

### 4.2 Analgesic efficacy comparison

Ultrasound guidance enables precise identification of fascial planes and neural structures, facilitating accurate anesthetic deposition in the iliofascial compartment. This technique effectively blocks nociceptive afferent transmission through preganglionic fibers, this technique enhances regional analgesia in the proximal femoral surgical field. The intervention demonstrates significant clinical benefits in mitigating postoperative stress responses, including pain, hypercoagulable states, and inflammatory cascades. Our meta-analysis of 14 RCTs demonstrated significantly lower VAS scores in the FICB group compared to the intravenous analgesia group at all postoperative time points (0.5–48 h; *p* < 0.05). These findings align with existing evidence: Usman et al. (54) reported reduced pain scores post-FICB after 30 min, and Agarwal et al. (55) observed earlier pain relief (within 20 min) in elderly patients. Shukla et al. (56) further corroborated sustained analgesia at 24 h. Our findings on UG-FICB’s rapid onset and technical feasibility resonate with recent evidence from Bauiomy et al. (12). In their double-blind RCT comparing PENG block versus suprainguinal FICB (S-FICB) in hip fracture patients: S-FICB achieved significantly lower positioning VAS and demonstrated faster performance times. Collectively, these data strengthen our conclusion that UG-FICB should be prioritized over intravenous opioids for early pain control in hip fractures. Mechanistically, FICB interrupts nociceptive signaling at peripheral nerves (femoral, lateral femoral cutaneous, and obturator), mitigating central sensitization risk, whereas systemic opioids solely attenuate pain perception without inhibiting peripheral nociceptive mediator release (55). The substantial heterogeneity (I^2^ = 94%) observed in VAS outcomes warrants careful consideration. Sensitivity analyses failed to definitively establish sources, but we identified some principal contributors through analyses: 1. Drug selection and dosing: Included studies utilized varying local anesthetics (ropivacaine, bupivacaine) at concentrations spanning 0.25–0.5%. Crucially, a dose–response relationship was evident: studies administering ropivacaine at 3 mg/kg (20 mL volume) demonstrated 30% lower pain scores than those using 1.5 mg/kg (15 mL) (57). 2. Injection approaches: Both suprainguinal (S-FICB) and infrainguinal techniques were employed. S-FICB provides more consistent blockade of the femoral, lateral femoral cutaneous, and obturator nerves due to proximal deposition cranial to the inguinal ligament, whereas infrainguinal blocks exhibit variable spread to the obturator nerve. 3. Block duration: Continuous catheter techniques [reported in 8 studies (22, 23, 30, 31, 41, 36–38)] provided prolonged analgesia compared to single-shot injections, affecting longitudinal VAS trajectories. 4. Fracture morphology: clinical disparities in fracture types (femoral neck vs. intertrochanteric) and surgical approaches (internal fixation vs. arthroplasty). 5. Comorbidity burden: Unreported ASA classifications (missing in 11/26 studies) obscured risk stratification; ASA III/IV patients likely experienced heightened pain sensitivity due to polypharmacy and reduced functional reserve. Collectively, these factors complicate cross-study comparisons. Future RCTs should standardize UG-FICB protocols (S-FICB approach, fixed anesthetic volumes) and stratify analyses by fracture type and mobility status to mitigate heterogeneity.

The FICB reduced postoperative opioid requirements by 5.27 folds and decreased total morphine milligram equivalents by 7.79 units (*p* < 0.00001), aligning with pharmacokinetic models of prolonged nociceptive blockade. Pissens et al. (58) demonstrated a 50% reduction in opioid demand post-FICB compared to intravenous fentanyl (*p* = 0.04), with superior patient satisfaction (*p* < 0.001). Similarly, Zhou et al. (59) reported enhanced analgesia and reduced analgesic requirements through triple nerve blockade (femoral, obturator, and lateral femoral cutaneous nerves), which sustains nociceptive inhibition for 12–24 h, delaying opioid initiation and cumulative dosing. In contrast, intravenous analgesia depends on drug half-lives (fentanyl’s 3–7-h duration), necessitating repeated dosing and resulting in plasma concentration fluctuations that exacerbate opioid demand (58). FICB shows the superiority in pain control, positioning tolerance, and satisfaction scores. The 3.8-fold improvement in patient satisfaction (OR = 0.26, *p* = 0.0002) observed in our study likely stems from rapid positional pain relief and reduced opioid-induced nausea/vomiting. Additionally, FICB shortened hospital stays, enhancing rehabilitation adherence—a finding consistent with Callear et al.’s report of reduced analgesic needs (*p* = 0.03) and expedited discharge readiness (60, 61). These outcomes underscore FICB’s dual role in opioid stewardship and accelerated recovery pathways.

### 4.3 Adverse effect profile

Compared to UG-FICB, intravenous analgesia significantly increased dizziness (OR = 2.34) and hypersomnia (OR = 3.58; both *p* < 0.05). Opioid-sparing effects and peripheral nociceptive blockade mitigated central inhibitory cascades. Activation of *μ*-receptors in the locus coeruleus and vestibular nuclei directly induced sedation and respiratory depression (62). Studies have demonstrated that sleep disorders (particularly those involving sleep duration, circadian rhythm sleep–wake disturbances, and sleep-disordered breathing) may elevate the risk of cognitive impairment and delirium, thereby adversely impacting disease prognosis (63). Despite these connections, delirium incidence remained comparable between groups (OR = 1.51, *p* = 0.14) contrasting with prior evidence on regional anesthesia benefits (64). Potential confounders included uncontrolled variables (e.g., preoperative dementia, age ≥75 years [3-fold delirium risk vs. 65–75 years]), and surgical stress-induced cytokine release (65).

The intravenous analgesia cohort demonstrated a 3.42-fold higher incidence of gastrointestinal complications compared to FICB, with significantly elevated risks of nausea (OR = 2.57) and vomiting (OR = 1.97). Mechanistically, Opioid analgesics exert their adverse effects by suppressing the release of neurotransmitters mediating gastric contractile coordination and modulation. This pharmacological inhibition manifests clinically as diminished peristaltic contractions and impaired gastric emptying, resulting in prolonged retention of ingested solids and liquids within the gastric lumen (66). Studies reveal that FICB not only reduces opioid-associated adverse drug reactions, including sedation, nausea, and vomiting, but also achieves sustained analgesic efficacy through targeted neural blockade. This dual mechanism attenuates opioid-induced gastrointestinal dysmotility, thereby lowering the incidence of postoperative gastrointestinal complications (67). Opioids suppress intestinal contractions and propulsive movements needed for normal bowel function. This reduced motility slows gut transit, allowing excess fluid absorption that hardens stool, leading to medication-induced constipation. This is consistent with our findings (68). Beyond gastrointestinal effects, UG-FICB demonstrated superior safety profiles for respiratory and thrombotic outcomes. By avoiding opioid use, FICB reduces hypoxic events—particularly critical for patients with COPD (comprising 30% of hip fracture populations), who face higher risks of hypoxemia-induced respiratory depression and mortality (16). Preserved cough ability and early mobilization significantly lower aspiration risks, a vital advantage for elderly patients, correlating with reduced mortality rates in geriatric hip fracture cases. Meanwhile, opioids activate platelets via P-selectin upregulation. FICB likely exerts protective effects by reducing opioid exposure, thereby lowering thrombus formation (69). Therefore, FICB is safer and more effective for elderly patients with hip fractures.

### 4.4 Current limitation

Our search was restricted to peer-reviewed journal articles. Grey literature (such as: conference proceedings, dissertations, trial registries) and unpublished studies were excluded due to challenges in verifying methodological rigor and data completeness. While methodologically justified for quality control, it may have omitted unpublished null/negative studies, particularly for outcomes with borderline fail-safe *N* values. Therefore, it risks omitting null/negative results, potentially introducing publication bias. Future searches of relevant grey literature databases should be conducted to enhance the comprehensiveness of this evidence base. Regarding heterogeneity, uncontrolled variables (block duration, adjunct medication regimens) constrain the generalizability of our findings. This meta-analysis revealed substantial heterogeneity (I^2^ = 94%), attributable to variability in FICB approach (suprainguinal vs. infrainguinal), inconsistent local anesthetic concentrations, and heterogeneity in baseline comorbidities and patient age. Inadequate randomization and concealment in 77% of studies may have skewed group allocation, particularly given UG-FICB’s technical demands. This could exaggerate analgesic benefits and underestimate adverse events. Lack of blinding in 77% of trials risks overestimating UG-FICB’s efficacy for subjective outcomes (VAS, satisfaction). Unblinded assessors may systematically underscore pain in UG-FICB groups, partly explaining the 94% heterogeneity in VAS results. Unreported dropout rates in 11 studies (42%) threaten result completeness, especially for delirium. Future research should prioritize large-scale randomized controlled trials incorporating longitudinal follow-up evaluating delirium incidence, cognitive trajectories, and patient-reported quality of life metrics is critical to delineate the impact of regional analgesia on functional recovery and survival outcomes.

## 5 Conclusion

This meta-analysis demonstrates that UG-FICB significantly enhances postoperative analgesia efficacy, reduces opioid-related adverse effects, and improves patient-reported satisfaction in geriatric hip fracture patients. However, UG-FICB’s applicability remains constrained by its dependency on specialized ultrasound equipment and operator expertise. Given the substantial heterogeneity observed in this meta-analysis (I^2^ = 94%), the pooled estimates should be interpreted with caution, as high heterogeneity reflects underlying clinical and methodological variations (such as: FICB techniques, anesthetic dosing, fracture types, and patient comorbidities) that may limit the certainty of the results. Future studies should standardize protocols to expand clinical utility, while multicenter, double-blinded, large-sample randomized controlled trials with extended follow-up periods are warranted to validate the sustained efficacy and safety outcomes.

## Data Availability

All data generated or analyzed during this study are included in this published article.

## References

[1] Sheehan KJ Williamson L Alexander J Filliter C Sobolev B Guy P . Prognostic factors of functional outcome after hip fracture surgery: a systematic review. Age Ageing. (2018) 47:661–70. doi: 10.1093/ageing/afy057 29668839

[2] Loggers SAI Van Lieshout EMM Joosse P Verhofstad MHJ Willems HC. Prognosis of nonoperative treatment in elderly patients with a hip fracture: a systematic review and meta-analysis. Injury. (2020) 51:2407–13. doi: 10.1016/j.injury.2020.08.027 32907702

[3] Zhang C Feng J Wang S Gao P Xu L Zhu J . Incidence of and trends in hip fracture among adults in urban China: a nationwide retrospective cohort study. PLoS Med. (2020) 17:e1003180. doi: 10.1371/journal.pmed.1003180 32760065 PMC7410202

[4] Brauer CA Coca-Perraillon M Cutler DM Rosen AB. Incidence and mortality of hip fractures in the United States. JAMA. (2009) 302:1573–9. doi: 10.1001/jama.2009.1462 19826027 PMC4410861

[5] Groff H Kheir MM George J Azboy I Higuera CA Parvizi J. Causes of in-hospital mortality after hip fractures in the elderly. Hip Int. (2020) 30:204–9. doi: 10.1177/1120700019835160 30909746

[6] Collin PG D'Antoni AV Loukas M Oskouian RJ Tubbs RS. Hip fractures in the elderly-: a clinical anatomy review. Clin Anat. (2017) 30:89–97. doi: 10.1002/ca.22779 27576301

[7] Lin J Yang Y Fei Q Zhang X Ma Z Wang Q . Validation of three tools for identifying painful new osteoporotic vertebral fractures in older Chinese men: bone mineral density, osteoporosis self-assessment tool for Asians, and fracture risk assessment tool. Clin Interv Aging. (2016) 11:461–9. doi: 10.2147/CIA.S101078 27217730 PMC4853018

[8] Zhang ZQ Ho SC Chen ZQ Zhang CX Chen YM. Reference values of bone mineral density and prevalence of osteoporosis in Chinese adults. Osteoporos Int. (2014) 25:497–507. doi: 10.1007/s00198-013-2418-2 23800746

[9] Motamed C. Clinical update on patient-controlled analgesia for acute postoperative pain. Pharmacy (Basel). (2022) 10:22. doi: 10.3390/pharmacy10010022 35202071 PMC8877436

[10] Mercadante S. Intravenous morphine for management of cancer pain. Lancet Oncol. (2010) 11:484–9. doi: 10.1016/S1470-2045(09)70350-X 20434717

[11] Kestenbaum MG Vilches AO Messersmith S Connor SR Fine PG Murphy B . Alternative routes to oral opioid administration in palliative care: a review and clinical summary. Pain Med. (2014) 15:1129–53. doi: 10.1111/pme.12464 24995406

[12] Bauiomy H Kohaf NA Saad M Abosakaya AM. Comparison between peri-capsular nerve group and supra inguinal fascia iliaca block for analgesia and ease of positioning during neuraxial anesthesia in hip fracture patients: a randomized double-blind trial. Egypt J Anaesth. (2024) 40:193–200. doi: 10.1080/11101849.2024.2333708

[13] Yang Z Xu W Xu S. Comparison of the efficacy and safety of different puncture routes for ultrasound-guided fascia iliaca compartment block for early analgesia after hip arthroplasty: a meta-analysis. Medicine (Baltimore). (2024) 103:e39313. doi: 10.1097/MD.0000000000039313 39213204 PMC11365671

[14] Li T Yan J Gao X Liu H Li J Shang Y . Using virtual reality to enhance surgical skills and engagement in orthopedic education: systematic review and meta-analysis. J Med Internet Res. 27:e70266. doi: 10.2196/70266 PMC12143859 40446337

[15] Li T Yan J Hu J Liu X Wang F. Efficacy and safety of electroacupuncture for carpal tunnel syndrome (CTS): a systematic review and meta-analysis of randomized controlled trials. Front Surg. (2022) 9:952361. doi: 10.3389/fsurg.2022.952361 36211261 PMC9539120

[16] Ali FM Ayub A Darlong V Pandey RK Punj J Sharma V. Ultrasound-guided suprainguinal fascia iliaca block to position the patient for neuraxial anaesthesia in acetabular surgery - a randomized controlled pilot study. Anaesthesiol Intensive Ther. (2024) 56:54–60. doi: 10.5114/ait.2024.138554 38741444 PMC11022634

[17] Bang S Chung J Jeong J Bak H Kim D. Efficacy of ultrasound-guided fascia iliaca compartment block after hip hemiarthroplasty: a prospective, randomized trial. Medicine (Baltimore). (2016) 95:e5018. doi: 10.1097/MD.0000000000005018 27684871 PMC5265964

[18] Chen T Zhou M Liu Y Liao X Tang L Zhang Y. Effeet of iliofascial block combined with lateral approach sciatie nerve blockand intravenous controlled analgesia on postoperative analgesiaafter lower limb fracture surgery. J Xingjiang Med Univ. (2023) 46:791–794+801. doi: 10.3969/j.issn.1009-5551.2023.06.015

[19] Guo J Ye J Yu L Sheng H Sun B Zhou X . Application of iliac fascial space block in treatment of elderly femoral intertrochanteric fractures. Chongqing Med. (2021) 50, 1531–1535. doi: 10.3969/j.issn.1671-8348.2021.09.021

[20] Han C Fan J. Effects of venlafaxine combined with FICB guided by ultrasound on postoperative cognitive function, serum 5-HT and NE levels in elderly patients with hip fracture. J Int Psychiatry. (2022) 49, 316–319. doi: 10.13479/j.cnki.jip.2022.02.044

[21] Khan MH Hussain S Khan MG Bahadar J Kashif M Uddin R. Efficacy of ultrasound guided fascia iliaca block for pain management compared to conventional pain killers; a comparative study. Pak J Med Health Sci. (2021) 15:2525–8. doi: 10.53350/pjmhs211582525

[22] Li C Ge W Yang K Zheng H Wang X Wang H . Ultrasound guided continuous fascia iliaca compartment block for perioperative pain management in elderly patients undergoing hip fracture surgery. Chin J Orthop Traumatol. (2023) 36:1046–51. doi: 10.12200/j.issn.1003-0034.2023.11.008 38012873

[23] Morrison RS Dickman E Hwang U Akhtar S Ferguson T Huang J . Regional nerve blocks improve pain and functional outcomes in hip fracture: a randomized controlled trial. J Am Geriatr Soc. (2016) 64:2433–9. doi: 10.1111/jgs.14386 27787895 PMC5173407

[24] Mostafa SF Eid GM Elkalla RS. Patient-controlled fascia iliaca compartment block versus fentanyl patient-controlled intravenous analgesia in patients undergoing femur fracture surgery. Egypt J Anaesth. (2018) 34:9–13. doi: 10.1016/j.egja.2017.12.002

[25] Ning J Wu A. The analgesic efficacy of fascia iliaca compartment block for pain control during changes of position in patients with inter-trochanteric fracture. Beijing Med. (2015) 37:564–6. doi: 10.15932/j.0253-9713.2015.6.021

[26] Piao H Wang R Zhao S Jin B. Effect of preemptive and multimodal analgesia on the rapid recovery of perioperative hip fracture in elderly patients. J Dalian Med Univ. (2020) 42:313–7. doi: 10.11724/jdmu.2020.04.06

[27] Qing L Qiu F Chen X. Effect of postoperative pain and complications on the B-ultrasound guided fascia iliaca compartment block for the elderly patients with proximal femur fracture. J Clin Orthop. (2018) 21, 215–220. doi: 10.3969/j.issn.1008-0287.2018.02.034

[28] Shan Z Huang H Li J Li H Wei D Yuan L. Effects of different analgesia regimens in patients with hip fracture during posture changing. Shanghai Med. (2020) 43:720–3. doi: 10.19842/j.cnki.issn.0253-9934.2020.12.003

[29] Shen Y Chen L. The effects of ultrasound-guided fascia iliaca compartment block on early analgesia in elderly patients with hip fracture. China J Mod Med. (2021) 31:37–42. doi: 10.3969/j.issn.1005-8982.2021.04.007

[30] Sun Q Yu J Chen Z. Effects of continuous fascial iliac compartment block on postoperative cognitive function and stress in elderly patients undergoing hip surgery. J Clin Anesthesiol. (2021) 37:603–6. doi: 10.12089/jca.2021.06.010

[31] Tan Z Zheng G Lu M Zhang L. Effect of continuous iliofascial space block on pain and stress response after hip fracture in elderly patients. Mod J Integr Trad Chin Western Med. (2019) 28:890–2. doi: 10.3969/j.issn.1008-8849.2019.08.023

[32] Thompson J Long M Rogers E Pesso R Galos D Dengenis RC . Fascia Iliaca block decreases hip fracture postoperative opioid consumption: a prospective randomized controlled trial. J Orthop Trauma. (2020) 34:49–54. doi: 10.1097/BOT.0000000000001634 31469752

[33] Wang J Cai N Zhang Z. Effect of FICB on intravertebral anesthesia in elderly patients with femoral neck fracture and femoral head replacement. Chin J Gerontol. (2023) 43:2097–100. doi: 10.3969/j.issn.1005-9202.2023.09.015

[34] Wang W Zhao J Han B Zhao L Dang X Li J . Application of continuous iliac fascial block guided by ultrasound in postoperative analgesia of senile intertrochanteric fracture. J Clin Ultrasound Med. (2020) 22, 77–78. doi: 10.3969/j.issn.1008-6978.2020.01.030

[35] Xie J Wang M Xia K Xu W Chen Y. Application of uitrasound-guided suprainguinal fascia iliaca compartment block in perioperative analgesia of elderly patients with hip fractures. Geriatr Health Care. (2024) 30:846–52. doi: 10.3969/j.issn.1008-8296.2024.03.051

[36] Xu T Liu Z Chen X Zhang S. Effect of continuous iliac fascial space block on perioperative analgesia, hemodynamics and postoperative delirium in advanced age patients undergoing hip surgery. Chin Med Herald. (2021) 18:101–4. doi: 10.20047/j.issn1673-7210.2021.34.023

[37] Xu X Liu H Kang C. Effect of continuous analgesia with iliofascial space block on postoperative pain and early recovery in elderly patients with hip fracture. World Clin Drug. (2023) 44, 488–492. doi: 10.13683/j.wph.2023.05.014

[38] Xu Z Zhang M Yang R Fu H Jiang Y Chang J. Effect of preoperative continuous fascia iliaca compartment block on perioperative sleep quality and postoperative delirium in elderly patients with hip fracture. J Clin Anesthesiol. (2020) 36:953–7. doi: 10.12089/jca.2020.10.004

[39] Yamamoto N Sakura S Noda T Nishiyama A Dan'ura T Matsui Y . Comparison of the postoperative analgesic efficacies of intravenous acetaminophen and fascia iliaca compartment block in hip fracture surgery: a randomised controlled trial. Injury. (2019) 50:1689–93. doi: 10.1016/j.injury.2019.03.008 30904248

[40] Yao F Shui Y Xiang J Yang B. Application of superior iliac fascia block of inguinal ligament combined with patient controlled intravenous analgesia in elderly patients after hip arthroplasty. China J Orthop Traumatol. (2024) 37:482–7. doi: 10.12200/j.issn.1003-0034.20220753 38778532

[41] Yao M Wu J Zhai W Wang Q Zhou L Liu J. Effect of continuous iliofascial space block on pain and adverse reactions in elderly patients with hip fracture in emergency room. Chin J Bone Joint Inj. (2019) 34. doi: 10.7531/j.issn.1672-9935.2019.06.013

[42] Maggi S Kelsey JL Litvak J Heyse SP. Incidence of hip fractures in the elderly: a cross-national analysis. Osteoporos Int. (1991) 1:232–41. doi: 10.1007/BF03187467 1790410

[43] Cooper C Cole ZA Holroyd CR Earl SC Harvey NC Dennison EM . Secular trends in the incidence of hip and other osteoporotic fractures. Osteoporos Int. (2011) 22:1277–88. doi: 10.1007/s00198-011-1601-6 21461721 PMC3546313

[44] Veronese N Maggi S. Epidemiology and social costs of hip fracture. Injury. (2018) 49:1458–60. doi: 10.1016/j.injury.2018.04.015 29699731

[45] Haentjens P Magaziner J Colón-Emeric CS Vanderschueren D Milisen K Velkeniers B . Meta-analysis: excess mortality after hip fracture among older women and men. Ann Intern Med. (2010) 152:380–90. doi: 10.7326/0003-4819-152-6-201003160-00008 20231569 PMC3010729

[46] Geizhals S Shou Y Rudnin S Tama M Greenstein J Hahn B . Femoral nerve blocks versus standard pain control for hip fractures: a retrospective comparative analysis. Clin Exp Emerg Med. (2024) 11:181–7. doi: 10.15441/ceem.23.112 38286508 PMC11237263

[47] Lee LA Caplan RA Stephens LS Posner KL Terman GW Voepel-Lewis T . Postoperative opioid-induced respiratory depression: a closed claims analysis. Anesthesiology. (2015) 122:659–65. doi: 10.1097/ALN.0000000000000564 25536092

[48] Duprey MS Dijkstra-Kersten SMA Zaal IJ Briesacher BA Saczynski JS Griffith JL . Opioid use increases the risk of delirium in critically ill adults independently of pain. Am J Respir Crit Care Med. (2021) 204:566–72. doi: 10.1164/rccm.202010-3794OC 33835902 PMC8491270

[49] Dalens B Vanneuville G Tanguy A. Comparison of the fascia iliaca compartment block with the 3-in-1 block in children. Anesth Analg. (1989) 69:705–13. doi: 10.1213/00000539-198912000-00003 2589650

[50] Guay J Kopp S. Peripheral nerve blocks for hip fractures in adults. Cochrane Database Syst Rev. (2020) 11:Cd001159. doi: 10.1002/14651858.CD001159.pub3 33238043 PMC8130997

[51] Foss NB Kristensen BB Bundgaard M Bak M Heiring C Virkelyst C . Fascia iliaca compartment blockade for acute pain control in hip fracture patients: a randomized, placebo-controlled trial. Anesthesiology. (2007) 106:773–8. doi: 10.1097/01.anes.0000264764.56544.d2 17413915

[52] Wennberg P Norlin R Herlitz J Sarenmalm EK Möller M. Pre-operative pain management with nerve block in patients with hip fractures: a randomized, controlled trial. Int J Orthop Trauma Nurs. (2019) 33:35–43. doi: 10.1016/j.ijotn.2018.11.003 30876869

[53] Ritcey B Pageau P Woo MY Perry JJ. Regional nerve blocks for hip and femoral neck fractures in the emergency department: a systematic review. CJEM. (2016) 18:37–47. doi: 10.1017/cem.2015.75 26330019

[54] Usman MA Rabiu MB Salahu D. A comparison of fascia iliaca compartment block with intravenous analgesia to improve pain control in patients with femoral fracture. J West Afr Coll Surg. (2024) 14:255–61. doi: 10.4103/jwas.jwas_66_23 38988430 PMC11232794

[55] Agarwal SB Kulshreshtha NK Mandloi P Dhomne N Jain T. Comparative study of intravenous fentanyl versus fascia iliaca compartment block in reducing pain for better position in fracture femur patients for sub arachnoid block. Int J Med Anesthesiol. (2021) 4:01–6. doi: 10.33545/26643766.2021.v4.i3a.270

[56] Shukla U Jahan M Naaz S Srivastava S. USG guided femoral nerve block vs fascia iliaca compartment block as post-operative analgesia in hip fracture patients. Int J Res Med Sci. (2018) 6:3057. doi: 10.18203/2320-6012.ijrms20183644

[57] Singh S Dhir S Marmai K Rehou S Silva M Bradbury C. Efficacy of ultrasound-guided transversus abdominis plane blocks for post-cesarean delivery analgesia: a double-blind, dose-comparison, placebo-controlled randomized trial. Int J Obstet Anesth. (2013) 22:188–93. doi: 10.1016/j.ijoa.2013.03.003 23648056

[58] Pissens S Cavens L Joshi G Bonnet M Sauter A Raeder J . Pain management after hip fracture repair surgery: a systematic review and procedure-specific postoperative pain management (PROSPECT) recommendations. Acta Anaesthesiol Belg. (2024) 75:15–31. doi: 10.56126/75.1.04

[59] Zhou Y Zhang WC Chong H Xi Y Zheng SQ Wang G . A prospective study to compare analgesia from femoral obturator nerve block with fascia Iliaca compartment block for acute preoperative pain in elderly patients with hip fracture. Med Sci Monit. (2019) 25:8562–70. doi: 10.12659/MSM.915289 31721757 PMC6873637

[60] Callear J Shah K. Analgesia in hip fractures. Do fascia-iliac blocks make any difference? BMJ Qual Improv Rep. (2016) 5. doi: 10.1136/bmjquality.u210130.w4147 PMC4752715 26893899

[61] Dangle J Kukreja P Kalagara H. Review of current practices of peripheral nerve blocks for hip fracture and surgery. Curr Anesthesiol Rep. (2020) 10:259–66. doi: 10.1007/s40140-020-00393-7

[62] Zhang Z Xu F Zhang C Liang X. Opioid mu-receptors in medullary raphe region affect the hypoxic ventilation in anesthetized rats. Respir Physiol Neurobiol. (2009) 168:281–8. doi: 10.1016/j.resp.2009.07.015 19632358 PMC3438222

[63] Chambers AM. The role of sleep in cognitive processing: focusing on memory consolidation. Wiley Interdiscip Rev Cogn Sci. (2017) 8. doi: 10.1002/wcs.1433 28044430

[64] Mouzopoulos G Vasiliadis G Lasanianos N Nikolaras G Morakis E Kaminaris M. Fascia iliaca block prophylaxis for hip fracture patients at risk for delirium: a randomized placebo-controlled study. J Orthop Traumatol. (2009) 10:127–33. doi: 10.1007/s10195-009-0062-6 19690943 PMC2744739

[65] Iamaroon A Wongviriyawong T Sura-Arunsumrit P Wiwatnodom N Rewuri N Chaiwat O. Incidence of and risk factors for postoperative delirium in older adult patients undergoing noncardiac surgery: a prospective study. BMC Geriatr. (2020) 20. doi: 10.1186/s12877-020-1449-8 PMC6998823 32013872

[66] Panchal SJ Müller-Schwefe P Wurzelmann JI. Opioid-induced bowel dysfunction: prevalence, pathophysiology and burden. Int J Clin Pract. (2007) 61:1181–7. doi: 10.1111/j.1742-1241.2007.01415.x 17488292 PMC1974804

[67] Li J Dai F Chang D Harmon E Ibe I Sukumar N . A practical analgesia approach to fragility hip fracture: a single-center, retrospective, cohort study on femoral nerve block. J Orthop Trauma. (2019) 33:175–9. doi: 10.1097/BOT.0000000000001391 30570615

[68] Müller-Lissner S Bassotti G Coffin B Drewes AM Breivik H Eisenberg E . Opioid-induced constipation and bowel dysfunction: a clinical guideline. Pain Med. (2017) 18:1837–63. doi: 10.1093/pm/pnw255 28034973 PMC5914368

[69] Sato K Adachi T Shirai N Naoi N. Continuous versus single-injection sciatic nerve block added to continuous femoral nerve block for analgesia after total knee arthroplasty: a prospective, randomized, double-blind study. Reg Anesth Pain Med. (2014) 39:225–9. doi: 10.1097/AAP.0000000000000076 24682080

