# Supplementary material for: Ultrasound-guided fascia iliaca compartment block versus intravenous analgesia in geriatric hip fractures: a systematic review and meta-analysis of randomized trials demonstrating superior pain control
Source: Front Med (Lausanne). 2025 Oct 14;12:1611618. doi: 10.3389/fmed.2025.1611618 (PMC12558913; doi:10.3389/fmed.2025.1611618)
Supplement: Supplementary file 1 [file Supplementary_file_1.docx]

Index and Keyword Terms Used in the Databases

| Databases | Search strategy |
| --- | --- |
| PubMed: | ((((((((((((((((((((((((((Fractures, Hip[Title/Abstract]) OR (Intertrochanteric Fractures[Title/Abstract])) OR (Fractures, Intertrochanteric[Title/Abstract])) OR (Subtrochanteric Fractures[Title/Abstract])) OR (Fractures, Subtrochanteric[Title/Abstract])) OR (Trochanteric Fractures[Title/Abstract])) OR (Fractures, Trochanteric[Title/Abstract])) OR (Trochlear Fractures, Femur[Title/Abstract])) OR (Femur Trochlear Fracture[Title/Abstract])) OR (Femur Trochlear Fractures[Title/Abstract])) OR (Fracture, Femur Trochlear[Title/Abstract])) OR (Fractures, Femur Trochlear[Title/Abstract])) OR (Trochlear Fracture, Femur[Title/Abstract])) OR (Femoral Trochlear Fractures[Title/Abstract])) OR (Femoral Trochlear Fracture[Title/Abstract])) OR (Fracture, Femoral Trochlear[Title/Abstract])) OR (Fractures, Femoral Trochlear[Title/Abstract])) OR (Trochlear Fracture, Femoral[Title/Abstract])) OR (Trochlear Fractures, Femoral[Title/Abstract])) OR (Femoral Fracture[Title/Abstract])) OR (Fracture, Femoral[Title/Abstract])) OR (Fractures, Femoral[Title/Abstract])) OR ("Femoral Fractures"[Mesh])) OR ("Hip Fractures"[Mesh])) AND (((((Fascia Iliaca Block[Title/Abstract]) OR (Fascia Iliaca Compartment Block[Title/Abstract])) OR (FICB[Title/Abstract])) OR (fascia iliaca block[Title/Abstract])) OR ("Fascia"[Mesh]))) AND ((((((((((((((((((((((((((Diagnostic Ultrasound[Title/Abstract]) OR (Diagnostic Ultrasounds[Title/Abstract])) OR (Ultrasound, Diagnostic[Title/Abstract])) OR (Ultrasounds, Diagnostic[Title/Abstract])) OR (Echography[Title/Abstract])) OR (Echotomography[Title/Abstract])) OR (Sonography, Medical[Title/Abstract])) OR (Medical Sonography[Title/Abstract])) OR (Ultrasonic Imaging[Title/Abstract])) OR (Imaging, Ultrasonic[Title/Abstract])) OR (Ultrasonographic Imaging[Title/Abstract])) OR (Imagings, Ultrasonographic[Title/Abstract])) OR (Imaging, Ultrasonographic[Title/Abstract])) OR (Ultrasonographic Imagings[Title/Abstract])) OR (Ultrasound Imaging[Title/Abstract])) OR (Imagings, Ultrasound[Title/Abstract])) OR (Imaging, Ultrasound[Title/Abstract])) OR (Echotomography, Computer[Title/Abstract])) OR (Computer Echotomography[Title/Abstract])) OR (Tomography, Ultrasonic[Title/Abstract])) OR (Ultrasonic Tomography[Title/Abstract])) OR (Diagnosis, Ultrasonic[Title/Abstract])) OR (Diagnoses, Ultrasonic[Title/Abstract])) OR (Ultrasonic Diagnoses[Title/Abstract])) OR (Ultrasonic Diagnosis[Title/Abstract])) OR ("Ultrasonography"[Mesh]))) AND (((((Aged, 80[Title/Abstract] AND over[Title/Abstract]) OR (Elderly[Title/Abstract])) OR (geriatric[Title/Abstract])) OR (older adult[Title/Abstract])) OR ("Aged"[Mesh])) |
| Cochrane database: | #1 [MeSH descriptor:Hip Fractures] OR (hip fracture* OR intertrochanteric OR subtrochanteric OR trochanteric OR femur trochlear OR femoral fracture*)  #2 [MeSH descriptor:Fascia] OR (fascia iliaca block OR FICB OR "compartment block")  #3 [MeSH descriptor:Ultrasonography] OR (ultraso* OR echogra* OR sono*)  #4 [MeSH descriptor:Aged] OR (geriatric OR elderly OR "80 and over")  #5 #1 AND #2 AND #3 AND #4 |
| Embase: | 1. exp hip fracture/ or (hip fracture* or intertrochanteric fracture* or subtrochanteric fracture* or trochanteric fracture* or femur trochlear fracture* or femoral fracture*).ti,ab,kw.  2. exp fascia iliaca block/ or (fascia iliaca block or fascia iliaca compartment block or FICB).ti,ab,kw.  3. exp ultrasonography/ or (ultrasound or ultrasonograph* or echograph* or sonograph*).ti,ab,kw.  4. exp aged/ or (geriatric or elderly or "80 and over" or octogenarian*).ti,ab,kw.  5. 1 and 2 and 3 and 4 |
| Web of Science: | TS=(  ("hip fracture*" OR "intertrochanteric fracture*" OR "subtrochanteric fracture*" OR "trochanteric fracture*" OR "femur trochlear fracture*" OR "femoral fracture*") AND ("fascia iliaca block" OR "FICB" OR "compartment block") AND (ultraso* OR echogra* OR sono*) AND (geriatric OR elderly OR aged OR "80 and over") |
| Scopus: | TITLE-ABS-KEY(("hip fracture" OR "intertrochanteric fracture" OR "subtrochanteric fracture" OR "trochanteric fracture" OR "femur trochlear fracture" OR "femoral fracture") AND ("fascia iliaca block" OR "FICB" OR "compartment block") AND (ultrasound OR ultrasonogra* OR echogra* OR sonogra*) AND (geriatric OR elderly OR aged OR "80 and over") |
| CNKI | 主题=(髂筋膜阻滞 OR 髂筋膜间隙阻滞 OR FICB) AND 主题=(髋部骨折 OR 股骨骨折 OR 转子间骨折) AND 主题=(超声 OR 超声引导) AND 主题=(老年人 OR 老年患者 OR 高龄) |
| VIP | (主题:("髂筋膜阻滞" OR "髂筋膜间隙阻滞" OR "FICB") ) AND (主题:("髋部骨折" OR "股骨骨折" OR "转子间骨折") ) AND (主题:("超声" OR "超声引导") ) AND (主题:("老年人" OR "老年患者" OR "高龄") ) |
| Wan Fang | (M=(髂筋膜阻滞 + 髂筋膜间隙阻滞 + FICB) ) *M=(髋部骨折 + 股骨骨折 + 转子间骨折) ) *M=(超声 + 超声引导) ) *M=(老年人 + 老年患者 + 高龄) ) |
