# Supplementary material for: Ultrasound-guided fascia iliaca compartment block versus intravenous analgesia in geriatric hip fractures: a systematic review and meta-analysis of randomized trials demonstrating superior pain control
Source: Front Med (Lausanne). 2025 Oct 14;12:1611618. doi: 10.3389/fmed.2025.1611618 (PMC12558913; doi:10.3389/fmed.2025.1611618)
Supplement: Supplementary file 2 [file Supplementary_file_2.docx]

Supplementary file 2: GRADE assessment of clinical outcomes

| Certainty assessment | | | | | | |  | Effect |  | Certainty |
| --- | --- | --- | --- | --- | --- | --- | --- | --- | --- | --- |
| Clinical outcomes | Study design | Risk of bias | Inconsistency | Indirectness | Imprecision | Other considerations |  | SMD/MD/OR (95% CI) |  |  |
| 0.5 h VAS scores | 4 RCTs | No serious | Very serious ^c^ | No serious | Very Serious ^e^ | No |  | 0.77 (0.11 to 1.43) |  | ⨁◯◯◯Very low |
| 2 h VAS scores | 6 RCTs | No serious | Serious ^b^ | No serious | Very Serious ^e^ | No |  | 0.54 (0.22 to 0.86) |  | ⨁◯◯◯Very low |
| 4 h VAS scores | 4 RCTs | No serious | Very serious ^c^ | No serious | Very Serious ^e^ | No |  | 2.38 (0.89 to 3.88) |  | ⨁◯◯◯Very low |
| 6 h VAS scores | 8 RCTs | No serious | Very serious ^c^ | No serious | Very Serious ^e^ | No |  | 1.31 (0.73 to 1.89) |  | ⨁◯◯◯Very low |
| 12 h VAS scores | 10 RCTs | No serious | Very serious ^c^ | No serious | Very Serious ^e^ | No |  | 1.54 (0.72 to 2.35) |  | ⨁◯◯◯Very low |
| 24 h VAS scores | 14 RCTs | Serious ^a^ | Very serious ^c^ | No serious | Very Serious ^e^ | No |  | 1.39 (0.86 to 1.93) |  | ⨁◯◯◯Very low |
| 48h h VAS scores | 12 RCTs | Serious ^a^ | Very serious ^c^ | No serious | Very Serious ^e^ | No |  | 1.39 (0.63 to 2.15) |  | ⨁◯◯◯Very low |
| Analgesic requirement rate | 6 RCTs | No serious | No serious | No serious | Serious ^d^ | No |  | 5.27 (3.25 to 8.53) |  | ⨁⨁⨁◯Moderate |
| Reduced analgesic dosage | 3 RCTs | No serious | No serious | No serious | Serious ^d^ | No |  | 7.79 (5.67 to 9.91) |  | ⨁⨁⨁◯Moderate |
| Satisfaction rate | 5 RCTs | Serious ^a^ | No serious | No serious | No serious | No |  | 0.26 (0.12 to 0.52) |  | ⨁⨁⨁◯Moderate |
| Dizzy rate | 9 RCTs | Serious ^a^ | No serious | No serious | No serious | No |  | 2.34 (1.30 to 4.20) |  | ⨁⨁⨁◯Moderate |
| Hypersomnia rate | 7 RCTs | Serious ^a^ | No serious | No serious | No serious | No |  | 3.58 (1.92 to 6.67) |  | ⨁⨁⨁◯Moderate |
| Delirium rate | 7 RCTs | Serious ^a^ | No serious | No serious | No serious | No |  | 1.51 (0.88 to 2.58) |  | ⨁⨁⨁◯Moderate |
| Nausea | 10 RCTs | Serious ^a^ | No serious | No serious | No serious | No |  | 2.57 (1.45 to 4.56) |  | ⨁⨁⨁◯Moderate |
| Vomiting | 7 RCTs | Serious ^a^ | No serious | No serious | No serious | No |  | 1.97 (1.00 to 3.87) |  | ⨁⨁⨁◯Moderate |
| Gastric discomfort | 14 RCTs | Serious ^a^ | No serious | No serious | No serious | No |  | 4.64 (3.03 to 7.11) |  | ⨁⨁⨁◯Moderate |
| Constipation | 2 RCTs | No serious | No serious | No serious | Serious ^d^ | No |  | 4.82 (1.34 to 17.39) |  | ⨁⨁⨁◯Moderate |
| Interoperative bleeding loss | 4 RCTs | No serious | No serious | No serious | Serious ^d^ | No |  | 0.45 (-0.79 to 1.70) |  | ⨁⨁⨁◯Moderate |
| Operative time | 10 RCTs | Serious ^a^ | No serious | No serious | Serious ^d^ | No |  | -0.08 (-0.21 to 0.05 |  | ⨁⨁◯◯Low |
| Length of stay | 13 RCTs | Serious ^a^ | Very serious ^c^ | No serious | No serious | No |  | 1.88 (1.09 to 2.67) |  | ⨁◯◯◯Very low |
| Respiratory depression | 3 RCTs | Serious ^a^ | No serious | No serious | Serious ^d^ | No |  | 7.11 (1.25 to 40.61) |  | ⨁⨁◯◯Low |
| Pulmonary infection | 4 RCTs | Serious ^a^ | No serious | No serious | No serious | No |  | 2.30 (1.14 to 4.68) |  | ⨁⨁⨁◯Moderate |
| Thrombosis rate | 8 RCTs | Serious ^a^ | No serious | No serious | No serious | No |  | 2.56 (1.53 to 4.28) |  | ⨁⨁⨁◯Moderate |
| Cardiovascular accident rate | 5 RCTs | Serious ^a^ | No serious | No serious | No serious | No |  | 1.48 (0.80 to 2.74) |  | ⨁⨁⨁◯Moderate |
| Pruritus rate | 3 RCTs | No serious | No serious | No serious | Serious^d^ | No |  | 3.29 (0.85 to 12.71) |  | ⨁⨁⨁◯Moderate |

Notes:

CI: confidence interval; OR: odds ratio; SMD: standardized mean difference.

Explanations:

a > 50% of trials received “High” risk of bias ratings (≥1 out of 6 dimensions in the Cochrane Risk of Bias tool);

b I^2^ between > 50% and ≤75% points in either direction;

c I^2^ >75% points in either direction;

d 95% CI of an SMD extends between > 0.2 and ≤0.5 points in either direction, 95% CI of an MD extends between > 2 and ≤5.0 points in either direction, 95% CI of an OR extends between > 5.0 and ≤10.0 points in either direction;

e 95% CI of an SMD extends >0.5 points in either direction, 95% CI of an MD extends >5.0 points in either direction, 95% CI of an OR extends >10.0 points in either direction;
