# Supplementary material for: Ultrasound-guided fascia iliaca compartment block versus intravenous analgesia in geriatric hip fractures: a systematic review and meta-analysis of randomized trials demonstrating superior pain control
Source: Front Med (Lausanne). 2025 Oct 14;12:1611618. doi: 10.3389/fmed.2025.1611618 (PMC12558913; doi:10.3389/fmed.2025.1611618)

**Figure S1：**A forest plot showing the analgesic dosage


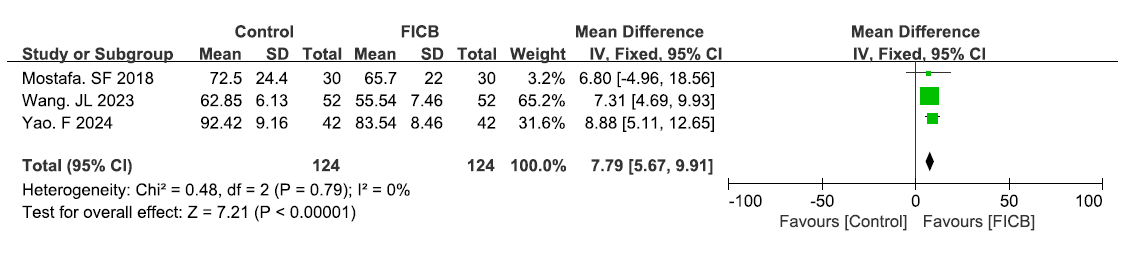


**Figure S2：**A forest plot showing the interoperative bleeding loss


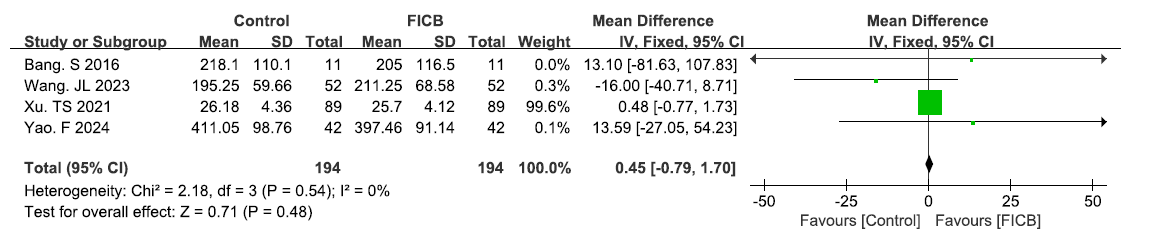


**Figure S3：**A forest plot showing the operative time


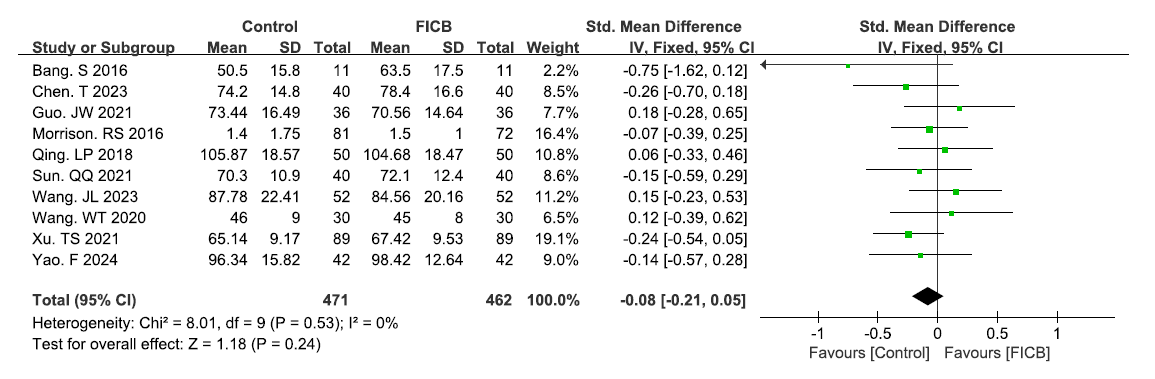


**Figure S4：**A forest plot showing the length of stay


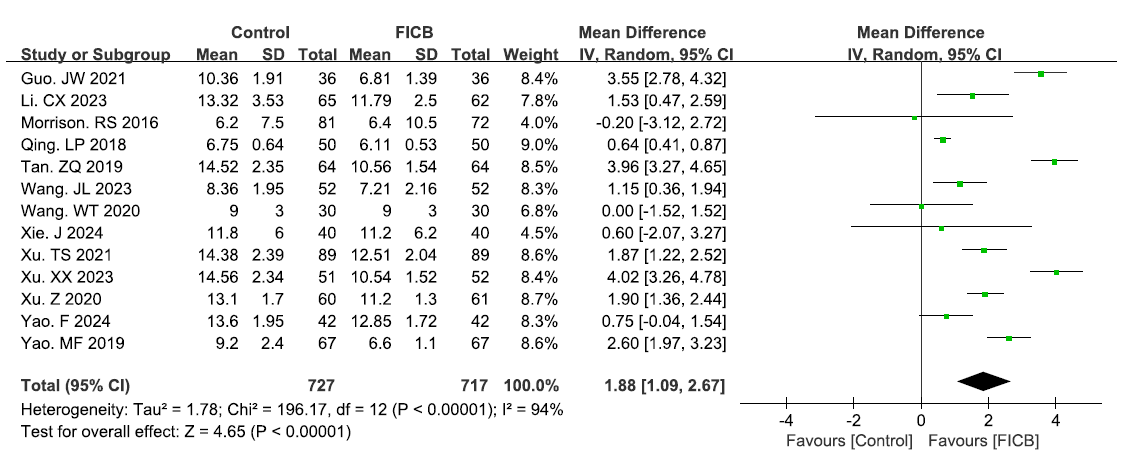


**Figure S5：**A forest plot showing the respiratory adverse


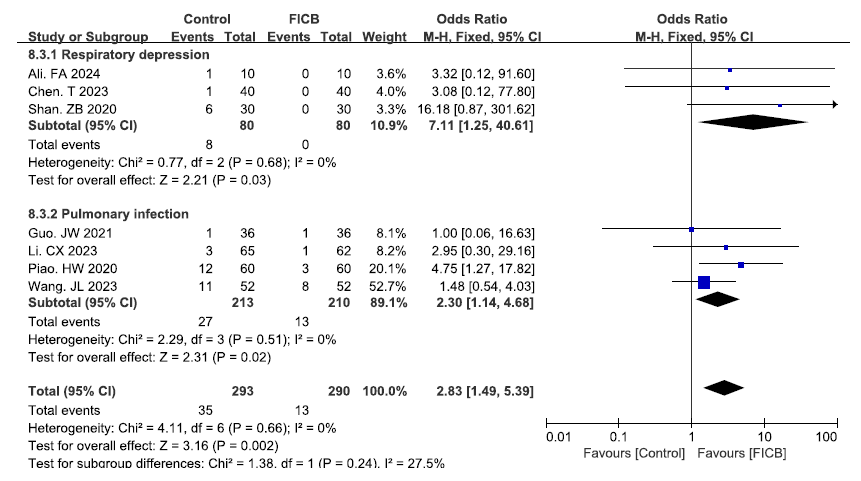


**Figure S6：**A forest plot showing the other adverse


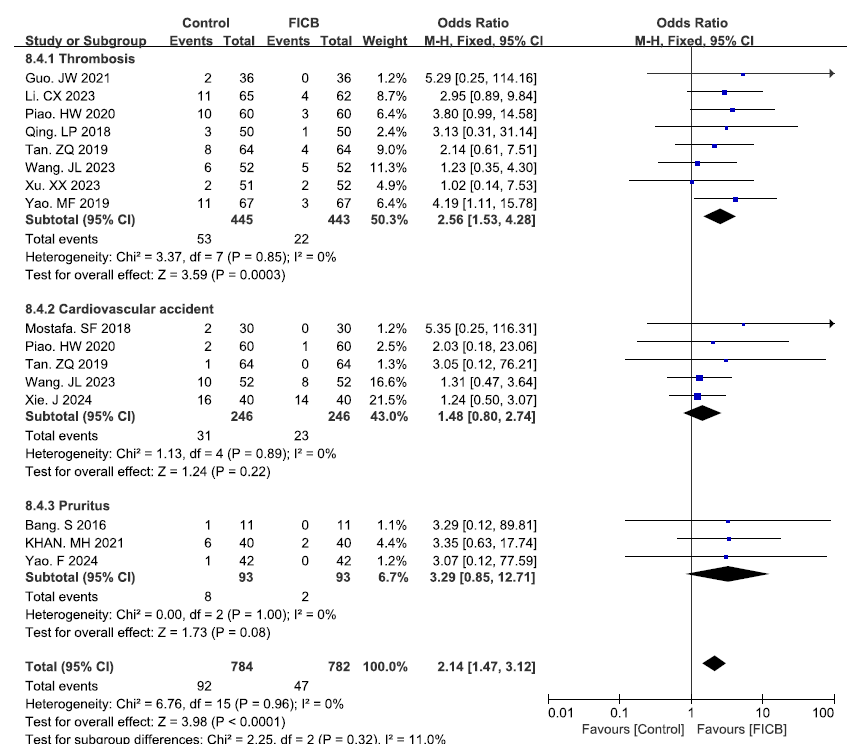


**Figure S7：**A plot showing the publication bias for length of stay


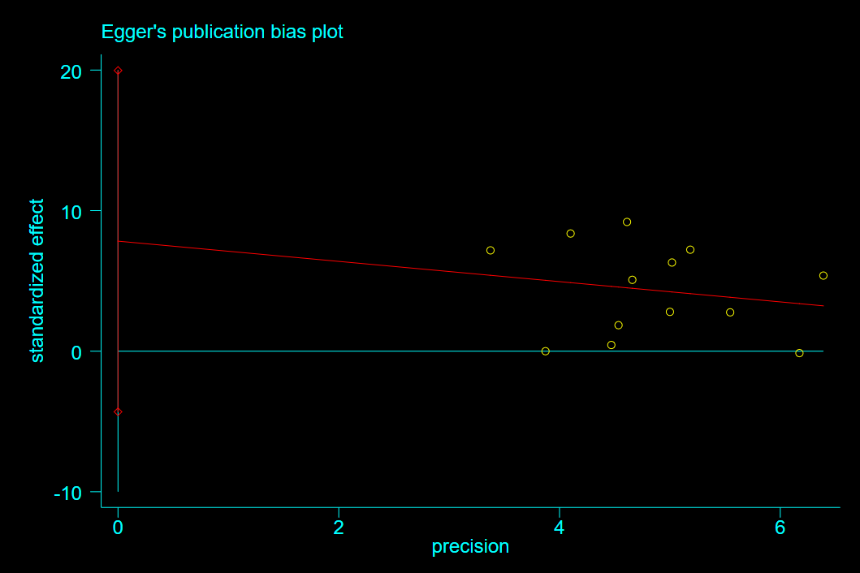


**Figure S8：**A plot showing the publication bias for operative time


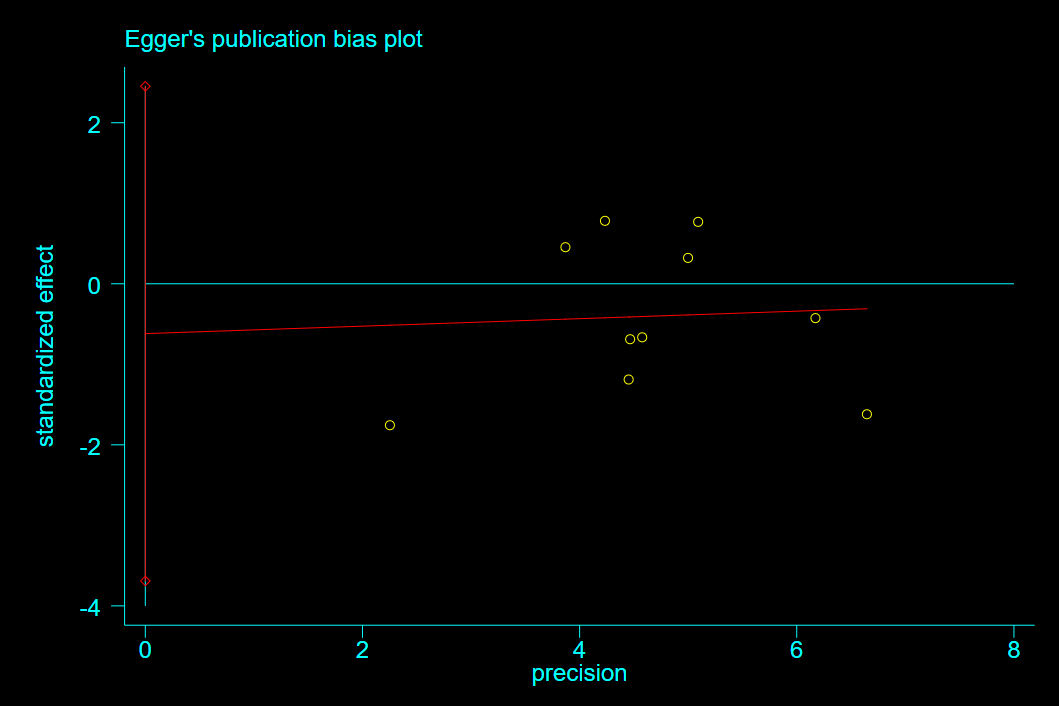

Supplement: Supplementary file 3 [file Supplementary_file_3.docx]
